# Formation and Properties of the Glassy Matrix: Vitrification Mechanism in *Haberlea rhodopensis* and the Resurrection Plants

**DOI:** 10.3390/ijms27219405

**Published:** 2026-10-22

**Authors:** Lyudmil Tonchev, Nikola Staykov, Tsanko Gechev

**Affiliations:** 1 Center of Plant Systems Biology and Biotechnology, Knyaz Boris I Pokrastitel 14, 4023 Plovdiv, Bulgaria; ltonchev@cpsbb.eu (L.T.); staykov@cpsbb.eu (N.S.); 2 Department of Molecular Biology, Faculty of Biology, University of Plovdiv, 24 Tsar Assen Str., 4000 Plovdiv, Bulgaria

**Keywords:** *Haberlea rhodopensis*, resurrection plants, desiccation tolerance, glass transition, LEA proteins, unfreezable water, natural deep eutectic solvents (NaDESs), cryotolerance, systems biology

## Abstract

Cytoplasmic vitrification is the standard explanation for how resurrection plants endure drying, yet the glass is rarely examined in tissue—its composition, glass transition and residual water remain unresolved. We characterize the glassy matrix of *Haberlea rhodopensis* by integrating transcriptomic and metabolomic data, biophysical modeling and targeted experiments. Sugar-metabolism genes, such as *SUCROSE-PHOSPHATE SYNTHASES* and *SUCROSE SYNTHASES*, respond to desiccation with strong concordance across three studies (Kendall’s W = 0.872; 81 genes measured in all three) and, with the metabolites, define a four-component glass-former set—sucrose plus the raffinose family oligosaccharides raffinose, stachyose and verbascose, with sucrose dominant (~92%). Apparent contradictions are plausibly explained: *INVERTASE* induction is consistent with compartmental separation from accumulating sucrose and declining trehalose with trehalose-6-phosphate (T6P) signaling, not vitrification. A parameterized state diagram traces the desiccation–rehydration path through vitrification and predicts that unfreezable water is locked throughout the glass. Desiccated tissue survived liquid nitrogen (−196 °C), suggesting that freezing tolerance reflects vitrification. Rewetting dried tissue was exothermic, consistent with an amorphous glass. We argue the residual water is functionally bound, that late embryogenesis abundant (LEA) proteins help shape the glass, and hypothesize that natural deep eutectic solvent (NaDES)-forming metabolites provide a transient reaction medium.

## 1. Introduction

Throughout their growth and development, plants are exposed to a wide range of stresses, which can be classified as biotic, caused by pathogens and herbivores, or abiotic, such as drought, salinity, extreme temperatures, and nutrient deficiency [1,2]. Among the abiotic stresses, drought is one of the most damaging: it limits growth, photosynthesis and reproduction [3], reduces national cereal production by about 10% in drought years [4], and alone causes greater annual crop yield losses than all pathogens combined [5]. Together with salinity and extreme temperatures, it also shapes the geographic distribution of plants and threatens food security [2], a problem that climate change is expected to intensify [3,5].

Plants counter water deficit through a coordinated network of responses. Drought is perceived and transduced through abscisic acid-dependent and -independent signaling pathways that activate transcription factors of the bZIP (AREB/ABF), AP2/ERF (DREB), NAC, MYB and WRKY families [6,7]. These regulate functional genes encoding protective proteins such as late embryogenesis abundant (LEA) proteins and heat shock proteins, enzymes for the synthesis of osmolytes and soluble sugars, and components of antioxidant systems [7]. Members of the WRKY family, for example, enhance tolerance to salinity and iron stress [8] and promote sucrose accumulation [9], while epigenetic mechanisms such as DNA methylation add a further layer of regulation to stress-related and developmental responses [10,11]. In most plants, however, these mechanisms confer tolerance only to moderate water loss. Resurrection plants are a striking exception: they can lose more than 95% of the water in their vegetative tissues and regain full function on rehydration [12,13]. This makes them a unique system for studying extreme desiccation tolerance and a source of genes for improving the stress tolerance of crops [12]. Understanding such a multilevel trait requires an integrative, systems-level approach.

Plant systems biology is a relatively recent, multidisciplinary field. Rather than isolating single genes or metabolic pathways, this approach integrates high-throughput multi-omics data, including genomics, transcriptomics, proteomics and metabolomics, with advanced computational modeling to map the complex, interconnected networks inherent in plants. Such integration is particularly revealing in resurrection plants, whose capacity to endure extreme water loss spans molecular, metabolic and biophysical scales.

Molecular studies of resurrection plants are abundant, but their desiccation tolerance has rarely been framed as a problem in solid-state biophysics. Vitrification is offered as the explanation almost by default, while the properties that would actually define the glass, such as its glass-transition temperature (T_g_), molecular mobility, and fragility, are rarely characterized in the living tissue. Vitrification is the arrest of a liquid into a glass: as it cools or concentrates, its viscosity climbs so steeply that molecular rearrangement halts and the disordered, liquid-like structure is locked in place as a rigid amorphous solid, without ever crystallizing [14]. To our knowledge, no single study of resurrection plants has brought together the composition of the glass-forming building blocks and their biophysical response to environmental change and progressive water loss in order to explain the mechanism of desiccation tolerance in these plants.

Soluble sugars are central to this mechanism. Sucrose, regarded as an important protectant in orthodox seeds, also accumulates in the drying leaves of resurrection plants as part of a protective program shared with maturing seeds [15,16,17,18]. In many species, it is accompanied by raffinose family oligosaccharides (RFOs), whose proportion relative to sucrose varies widely between species [16]. On drying, these non-reducing sugars, together with proteins and other solutes, form a highly viscous intracellular glass that slows molecular motion and is indispensable for survival in the dry state [19,20,21]. Which sugars a given plant produces specifically to build its glass, however, has rarely been determined.

The same metabolite may serve different functions in different organisms, so the composition of the intracellular glass cannot be inferred from other systems and must be established species by species. Here, we constructed a supplemented state diagram of the *Haberlea rhodopensis* sugar system and its vitrification strategy as a step toward that characterization. We first integrated the available transcriptomic and metabolomic datasets for *Haberlea* into a single comparative framework. Because sugars are regarded as the principal glass-formers, we analyzed published data on both their accumulation and the transcription of sugar-metabolism genes between the hydrated and desiccated states, comparing the two to identify the sugars most likely to contribute to vitrification, and used these to parameterize the diagram. Using *Haberlea* as a model, we further asked whether the residual water retained after drying has a function in resurrection plants or is solely a remnant of the dehydration process. We then extended the analysis to other molecules of interest, including the LEA proteins, which are thought to act both as molecular shields and as contributors to glass formation. In addition, we identified candidate natural deep eutectic solvent (NaDES)-forming metabolites and propose that they could assemble into a localized liquid phase serving as an alternative reaction medium as bulk water is lost from the cells. Finally, we tested selected predictions arising from the computational and literature-derived analyses in targeted laboratory experiments, which provide independent support for some of the theoretical results and permit additional conclusions to be drawn.

## 2. Results

### 2.1. Glassy State: Resurrection Plants vs. Orthodox Seeds

The parallel between orthodox seeds and resurrection plants is well established. Both survive almost complete water loss, and vegetative desiccation tolerance is widely interpreted as a redeployment in leaves of the protective program that operates during seed maturation. This comparison has been developed in detail at the molecular and physiological level: shared transcription factors and *LEA* gene families, comparable antioxidant and photosynthetic responses, and a similar accumulation of sucrose and RFOs [15,16,17,18]. What has not been compared is the physical state that these molecules produce. In seeds and pollen, the intracellular glass has long been treated as a material in its own right, with T_g_, molecular mobility and fragility measured and related to survival in the dry state [19,20,21]. In resurrection plants, the glassy state is invoked far more often than it is examined: vitrification is routinely offered as the explanation for desiccation tolerance, while the properties of the glass itself remain largely undescribed. The tissue most often compared to a seed has therefore rarely been compared to it on the property that vitrification is meant to explain. Here we address that asymmetry, setting the glassy matrix of *Haberlea rhodopensis* against what is known of the glass in orthodox seeds, and asking whether the same physical description holds.

The glassy state of the seed cytoplasm is among the better-supported ideas in desiccation biology, resting on several independent lines of evidence. Calorimetry detects a step in heat capacity (C_p_) at a T_g_ that falls predictably as water content rises [22,23]. Water acts as a plasticizer, and its depression of T_g_ is well described by the Gordon–Taylor equation, which treats the cytoplasm as a binary sugar–water mixture—the same formalism applied to synthetic polymer–diluent systems [24,25]. Spin-probe electron paramagnetic resonance (EPR) shows cytoplasmic rotational mobility slowing by orders of magnitude on drying, with the transition coinciding with the calorimetric T_g_ [26,27]. Seed deterioration follows the temperature dependence expected of a glass above T_g_ and slows sharply below it, which accounts for the extreme longevity of cool, dry seeds [28,29]. Fourier-transform infrared spectroscopy (FTIR) locates the transition in the molecules themselves: the O–H bands of sugars, the C–H bands of membrane lipids and the protein amide I band all shift on drying, and the resulting spectra match those of purified sucrose and trehalose glasses [30,31]. This chemical identification is consistent with composition—non-reducing di- and oligosaccharides accumulate in desiccation-tolerant tissues, the seed glass behaves as a complex oligosaccharide mixture, and engineering otherwise desiccation-sensitive cells to accumulate trehalose confers a degree of desiccation tolerance [25,32]. By contrast, desiccation-sensitive (recalcitrant) seeds lose viability at water content well above that at which a glassy state forms [19,32]. No single observation is decisive, but together they establish the seed cytoplasm as a sugar-based glass whose properties can be measured [19]. By contrast with the seed literature, the glassy state has rarely been determined or examined in resurrection plants. Using the FTIR approach described above, a glass transition was detected in dried leaves of *Craterostigma plantagineum*, with a T_g_ of about 65 °C that decreased with increasing water content. Moreover, the T_g_ of the dried cytoplasm exceeded that of a pure sucrose glass, indicating that sucrose is not the only component contributing to the formation of the glass [31].

### 2.2. Haberlea Sugar Matrix Composition

The sugars that participate in vitrification are not produced by any single biosynthetic route common to all resurrection plants. Each species accumulates its own combination, and it is this species-specific complement that forms the basis of the glass [33].

Chromatographic measurements are indispensable, but they do not yield absolute values. Differences in instrumentation and in sample preparation can produce discrepancies between studies. Combining several studies, several analytical methods and transcriptomic data therefore offers a firmer basis for interpretation than any single measurement.

Sugars are the principal building blocks of the glassy matrix in both resurrection plants and orthodox seeds. To estimate the sugar composition of the glassy matrix formed in *Haberlea*, we analyzed transcriptomic and metabolite data from several independent studies [34,35,36,37].

**Figure 1:**
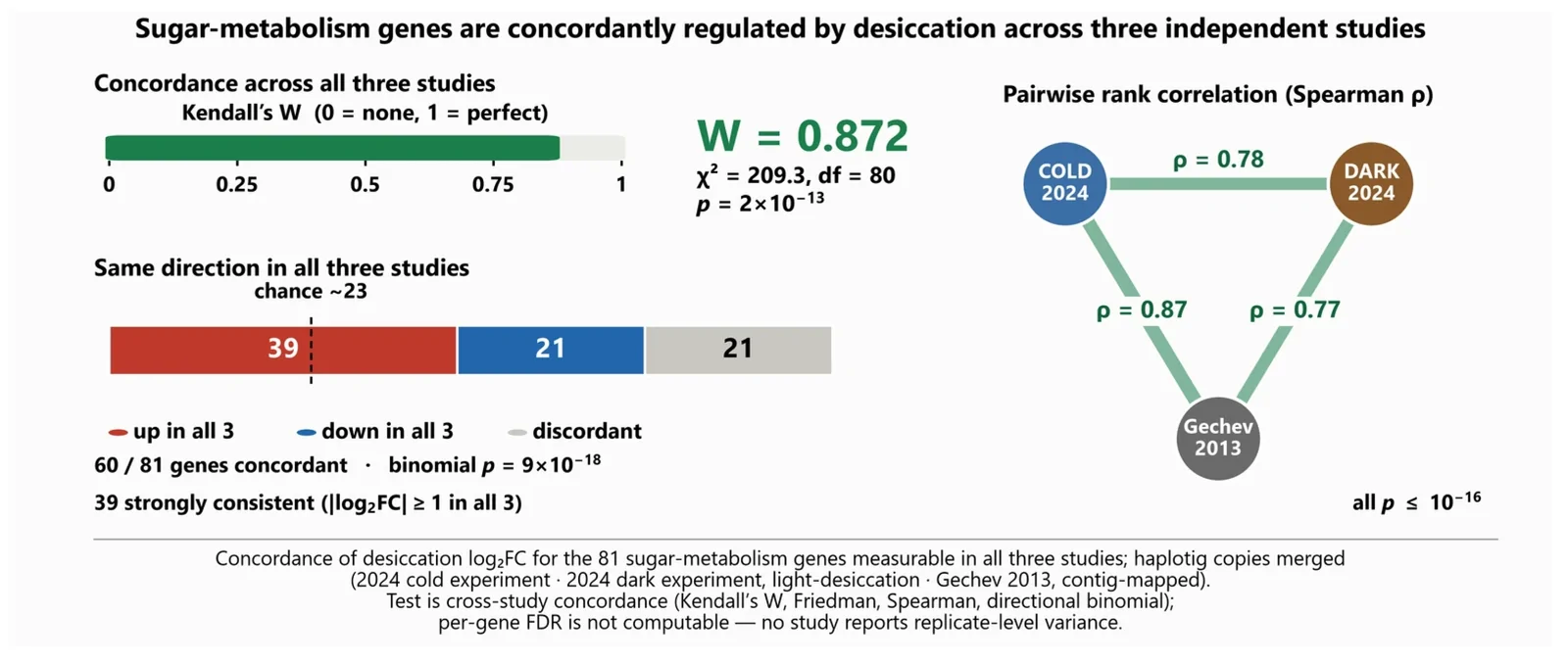
Sugar-metabolism genes are concordantly regulated by desiccation across three independent transcriptome studies. Overall agreement across the 81 sugar-metabolism genes (haplotig copies merged; Section 4.2) with a computable fold change in all three datasets (2024 cold experiment and 2024 dark experiment [35]; Gechev 2013 [34], contig-mapped) is quantified by Kendall’s coefficient of concordance (W = 0.872; χ^2^ = 209.3, df = 80, *p* = 2 × 10^−13^), directional consistency (60/81 genes change in the same direction in all three studies—39 up, 21 down—vs. ~23 expected by chance; binomial *p* = 9 × 10^−18^; 39 strongly consistent, |log_2_FC| ≥ 1), and pairwise Spearman rank correlations (ρ = 0.77–0.87, all *p* ≤ 10^−16^). Per-gene false-discovery rate (FDR) is not computable as no source study reports replicate-level variance. Because co-regulated genes are not fully independent, these *p*-values are anti-conservative and are best interpreted alongside the effect sizes (W and the 60/81 directional agreement), which do not depend on that assumption; a class-level analysis is given in Table S1.

**Figure 2:**
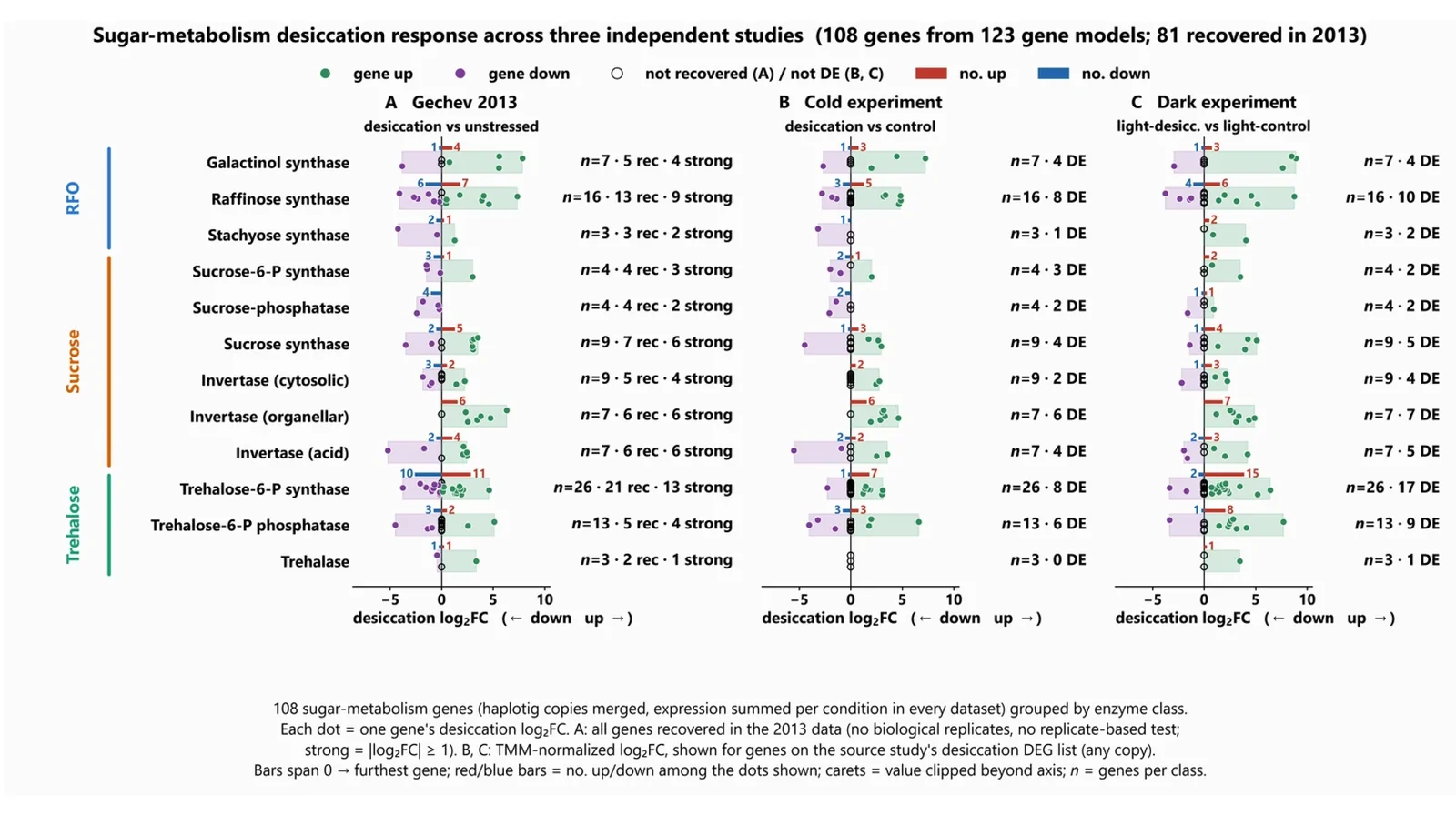
Desiccation response of the 108 sugar-metabolism genes (123 gene models; haplotig copies merged) across three independent studies, grouped by enzyme class and bracketed by pathway (RFO, sucrose, trehalose). Each dot is one gene’s desiccation log_2_ fold change: (**A**) Gechev 2013 [34], desiccation vs unstressed, all 81 genes recovered in the contig-mapped dataset (no biological replicates, hence no replicate-based significance test; strong = |log_2_FC| ≥ 1); (**B**) 2024 cold experiment [35], desiccation vs. control, and (**C**) 2024 dark experiment [35], light-desiccation vs. light-control, shown for genes on the source study’s list of desiccation-responsive differentially expressed genes (DEGs; log_2_FC of summed copies, normalized by the trimmed mean of M-values, TMM). Green, up-regulated; purple, down-regulated; open circles at 0, not recovered (**A**) or not differentially expressed (**B**,**C**); red and blue bars count the up- and down-regulated genes shown per class; carets mark values clipped beyond the axis; *n* = genes per class.

To cross-validate the transcriptomic data, the sugar-metabolism genes were compared across three studies [34,35]. The two 2024 transcriptomes were used only to test whether the transcriptional response is reproducible across independent experiments; the gene-level analysis of sugar metabolism in the following sections is based on the 2013 transcriptome, which was obtained from the same plants and at the same sampling time points as the 2013 metabolome, so gene expression and sugar accumulation are compared within one experiment (genes are counted as up- or down-regulated by the direction of their desiccation log_2_ fold change; gene models representing the same gene (haplotig copies) are merged and counted as one gene, Section 4.2). Although the studies did not follow strictly identical desiccation protocols, they showed significant concordance across the gene set (Figure 1), and gene expression followed a largely similar pattern (Figure 2); the per-gene cross-study mapping is provided in Table S1. The concordance proved robust to the non-independence of co-regulated genes (with each of the 12 enzyme classes treated as a single unit, W = 0.77, permutation *p* = 2 × 10^−4^; gene-level W = 0.872, cluster-bootstrap 95% confidence interval (CI) 0.80–0.91) to the stringency of the 2013 contig mapping (W = 0.86–0.88 when only 2013 contigs mapped at ≥95%, ≥98% or 100% identity were used, or when restricted to reciprocal best hits) and to the merging of haplotig copies (W = 0.873 for the 91 unmerged gene models; Table S1).

**Figure 3:**
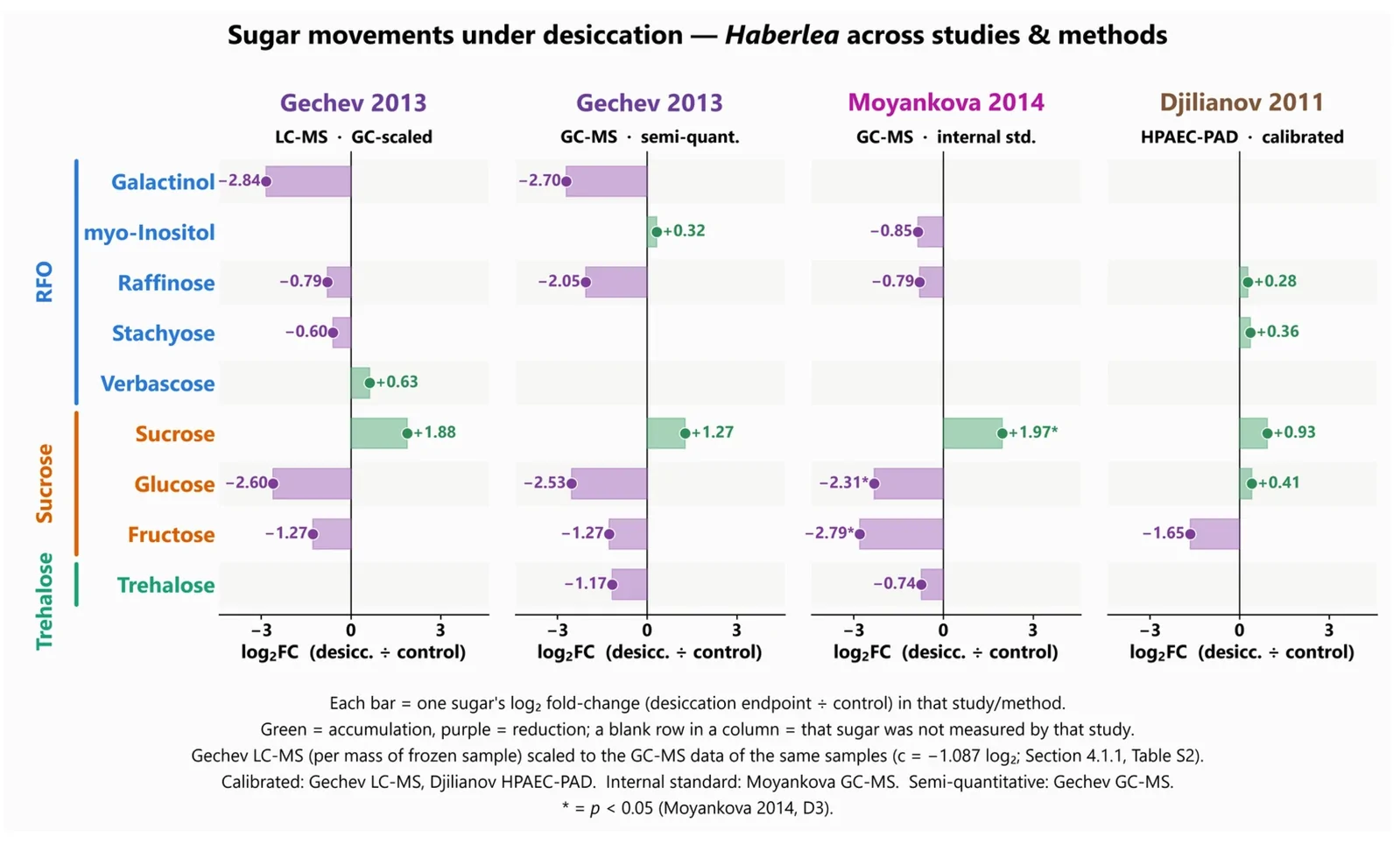
Sugar movements under desiccation in *Haberlea* across studies and analytical methods. Each bar is one sugar’s log_2_ fold change (desiccation endpoint ÷ control) in a given study/method, grouped by pathway (RFO, sucrose, trehalose). Columns: Gechev 2013 [34] (LC–MS, calibrated; fold changes rescaled to the GC–MS data of the same samples, Section 4.1.1) and Gechev 2013 (GC–MS, semi-quantitative), both desiccation (T3) vs. unstressed control (T1); Moyankova 2014 [36] (GC–MS, quantified against an internal standard), fully desiccated leaves (D3, relative water content (RWC) about 6%) vs. hydrated control; Djilianov 2011 [37] (HPAEC–PAD, calibrated), 168 h of drying (RWC 12%) vs. the 0 h control. Green, accumulation; purple, reduction; a blank row indicates the sugar was not measured by that study. After rescaling of the LC–MS fold changes, raffinose declines in three of the four datasets. * *p* < 0.05 (Moyankova 2014).

Before the platforms can be compared, the metabolite data have to be placed on a common basis. In the Supplementary Data of the 2013 study [34], the liquid chromatography–mass spectrometry (LC–MS) sugar values are given per mass of the frozen sample, without the corresponding dry weights, whereas the gas chromatography–mass spectrometry (GC–MS) values are normalized to dry weight. Because desiccated tissue contains far less water, values per mass of the frozen sample are higher in desiccated relative to hydrated tissue. To compare the two platforms on the same basis, we rescaled the LC–MS fold changes to the GC–MS data of the same samples, using a single factor derived from the five sugars measured by both methods (Section 4.1.1; Table S2). After this rescaling, the LC–MS and GC–MS data agree in the direction of change, as reported in the original study [34], and most sugars change in the same direction across studies and methods: sucrose rises in all datasets, glucose and fructose mostly decline, and raffinose declines in three of the four datasets, with only a slight increase by high-performance anion-exchange chromatography with pulsed amperometric detection (HPAEC–PAD) [37]. Because neither GC–MS dataset reported the higher RFOs, whose analysis by gas chromatography requires laborious derivatization [38], we based the sugar composition on the LC–MS data; as a ratio within the same samples, the composition does not depend on the weight basis. The comparison across studies is shown in Figure 3.

#### 2.2.1. Raffinose

Desiccation strongly induces the RFO biosynthetic pathway in *Haberlea*: four *GALACTINOL SYNTHASE* genes and seven *RFO-SYNTHASE (GALACTINOL–SUCROSE GALACTOSYLTRANSFERASE)* genes are up-regulated under desiccation. Because *RAFFINOSE SYNTHASES* and *STACHYOSE SYNTHASES* are close paralogues within *GLYCOSIDE HYDROLASE FAMILY 36 (GH36)* [39] and are assigned solely on the basis of sequence similarity, the specific reaction product of each induced gene cannot be deduced from its annotation alone.

Were all seven induced enzymes to synthesize raffinose from the available galactinol pool, raffinose would be expected to accumulate to high levels. This is not observed: in the 2013 study raffinose declines (0.58-fold by GC-scaled LC–MS and 0.24-fold by GC–MS), despite the pronounced transcriptional induction and a concurrent depletion of galactinol, the galactosyl donor. Three explanations are consistent with these data. First, metabolic flux—raffinose is synthesized, but immediately consumed by downstream reactions, so its steady-state pool never rises. Second—mis-annotation—a proportion of the induced “*RAFFINOSE SYNTHASE*” genes in fact encode stachyose or verbascose synthases, which use raffinose as a substrate rather than producing it [40]. Third, transcript abundance need not translate into enzyme activity: post-transcriptional or post-translational regulation could limit the amount or activity of the induced enzymes, as neither protein levels nor raffinose synthase activity were measured. The first two scenarios are consistent with the pattern of the higher-order RFOs: whereas raffinose and stachyose decline (0.58- and 0.66-fold, GC-scaled LC–MS), verbascose increases modestly (1.55-fold), as expected if raffinose is channeled onward into longer-chain RFOs rather than accumulating in its own right. Because the higher RFOs are minor components quantified only by LC–MS, this interpretation remains tentative, although the verbascose increase persists under all scaling factors tested except the largest offset, that of raffinose (Table S2).

#### 2.2.2. Myo-Inositol

As a low-abundance metabolic intermediate that feeds galactinol and RFO biosynthesis and is present at only ~1% of the sucrose and raffinose pools, myo-inositol is unlikely to be a major constituent of the protective sugar matrix.

#### 2.2.3. Stachyose and Verbascose

Although stachyose is an intermediate en route to verbascose, it possesses RFO-specific properties, and the three RFO sugars are almost invariably treated as a set in the literature. Data on verbascose remain comparatively scarce, particularly regarding the physical properties of this RFO. Drawing on established physicochemical principles together with the available studies on verbascose, several noteworthy inferences can be made about its accumulation and presence in resurrection plants. Collectively with the other RFOs, verbascose contributes to seed vigor and to protection against extreme-temperature stress by stabilizing proteins and membranes [41]. Consistent with this, seed vigor correlates not with the absolute amount of any single RFO—verbascose alone, for instance, but with the total RFO pool and its ratio to sucrose, indicating that these oligosaccharides act as a functional set rather than individually [42]. Its presence and increase during drying have been reported in some resurrection plants [33].

Once verbascose reaches a supersaturated state, its large molecular volume markedly raises the viscosity of the surrounding liquid, slowing molecular mobility and impeding the spontaneous nucleation of crystals. Rather than forming the rapid, well-ordered crystalline lattices characteristic of disaccharides such as sucrose, it tends instead to yield a dense, highly viscous syrup or an amorphous precipitate [43,44,45,46,47].

Taken together, these considerations suggest that despite their low relative abundance, verbascose and stachyose are not incidental by-products, but constituents of the protective sugar–glass matrix, on the strength of their distinctive physicochemical properties, the induction of the RFO-biosynthetic pathway (transcriptomic data), and the modest desiccation-associated rise in verbascose (GC-scaled LC–MS data).

#### 2.2.4. Sucrose

The signal shared by every dataset is the strong accumulation of sucrose during desiccation. In the Gechev 2013 study [34], sucrose rises 3.7-fold by GC-scaled LC–MS and 2.4-fold by GC–MS—the largest increase among the quantified sugars, consistent with the 3.9-fold increase in an independent dataset [36]. The increase of sucrose, observed at metabolite level during dehydration, is further corroborated by the strong induction of a *SUCROSE-PHOSPHATE SYNTHASE* gene (Hrh_00398), encoding the key enzyme of sucrose synthesis, as also reported by Gechev et al. [34]. Likewise, genes involved in sucrose utilization and relocation, such as *SUCROSE SYNTHASES* and a *SUCROSE TRANSPORTER*, are among the highly induced during dehydration and desiccation [34]. Invertase, the enzyme that hydrolyzes sucrose, is among the most strongly induced classes of sucrose metabolism. Of the sixteen neutral/alkaline *INVERTASE* genes, eleven are detected in the 2013 data, and eight of these are up-regulated under desiccation (three down). Taken at face value, the plant appears to accumulate and degrade the same compound at once. The metabolite data, however, are unambiguous: sucrose accumulates rather than declines, so no net hydrolysis occurs, and the induction must be reconciled with the accumulation. Invertases, however, do not form a single pool: the family comprises cytosolic, organellar and vacuolar/cell-wall isoforms that act on physically separate sucrose pools. A plausible resolution therefore emerges once the invertases are considered by subcellular compartment and by transcript abundance rather than by gene count alone. The organellar (chloroplast/mitochondrial) neutral invertases form the core of the response: all six detected genes are strongly and uniformly induced (log_2_FC + 2.3 to +6.3; ~7-fold net increase in summed, depth-normalized transcript). These enzymes are confined to the plastid and mitochondrion [48] and are therefore unlikely to deplete the bulk cytosolic and vacuolar sucrose pool that accumulates. The cytosolic induction is real, but mixed: two isoforms are strongly up-regulated (Hrh_06981, +1.4; Hrh_34318, +2.2), whereas the most highly expressed cytosolic isoform, Hrh_31754, is repressed (−1.0), yielding a moderate ~1.8-fold net increase in cytosolic capacity. In particular, among the vacuolar/apoplastic (acid) invertases—the only class with access to stored sucrose—the response is dominated by the repression of the principal isoform: the most highly expressed acid invertase in unstressed tissue, the *SOLUBLE (VACUOLAR) β-FRUCTOFURANOSIDASE* Hrh_29920, is repressed ~37-fold (log_2_FC −5.2), and a second soluble isoform is likewise down (−1.7). Two minor vacuolar isoforms are induced, but total vacuolar acid-invertase transcript still falls ~3.6-fold; the main induced acid invertase is the cell-wall (insoluble) isoform Hrh_15971 (~4-fold), an apoplastic enzyme that acts on sucrose outside the cell rather than on the vacuolar store. The collapse of the dominant vacuolar enzyme thus removes the principal route to hydrolysis of the stored sucrose. Sucrose accumulation and *INVERTASE* induction are therefore not necessarily contradictory: the induction is concentrated in organellar enzymes spatially separated from the vacuolar sucrose pool and in apoplastic (cell-wall) isoforms, while the single dominant vacuolar invertase, the one enzyme positioned to hydrolyze the accumulating sucrose, is strongly repressed. This compartment-based explanation rests on transcript levels and on the subcellular localization of the closest characterized homologues; confirming it would require measurements of compartment-specific invertase activity and of vacuolar sucrose.

#### 2.2.5. Trehalose

Trehalose has well-documented protective properties in anhydrobiotic systems [49]. Some resurrection plants—*Selaginella*, for example—have adopted trehalose as a principal sugar in their vitrification strategy [33]. In *Haberlea*, by contrast, induction of specific trehalose-pathway genes is accompanied by a decline in trehalose itself during desiccation. Alongside a partial up-regulation of *TREHALOSE-6-PHOSPHATE SYNTHASE (TPS)*, two *TREHALOSE-6-PHOSPHATE PHOSPHATASE (TPP)* genes, encoding the enzymes that dephosphorylate trehalose-6-phosphate (T6P) to trehalose, are strongly induced (Hrh_22305, ~6-fold; Hrh_32736, more than thirty-fold), although three other *TPP* genes are down-regulated. In parallel, a *TREHALASE* gene is strongly up-regulated (Hrh_06682, +3.4 log_2_FC), providing a route for the immediate hydrolysis of any trehalose formed. The induction of trehalose-metabolism genes could represent a compensatory response to declining trehalose levels. However, with *TREHALASE* induced at the same time, such a response would not be expected to restore the trehalose pool. That trehalose nonetheless declines despite an active pathway indicates that flux through T6P is not directed toward trehalose accumulation. The significance of this pattern follows from the signaling role of T6P [50]. T6P serves as a proxy for cellular sucrose status: its concentration tracks that of sucrose, and one of its proposed actions, at least in growing tissues, is inhibition of SUCROSE NON-FERMENTING1-RELATED PROTEIN KINASE 1 (SnRK1), the central energy-deficit kinase. Under favorable conditions, a rising sucrose pool elevates T6P, represses SnRK1, and promotes growth, storage-compound synthesis and sucrose utilization. A desiccating plant requires the opposite mechanism. Sucrose must accumulate to protective, matrix-forming concentrations while growth is arrested and the SnRK1-dependent stress program stays engaged. The transcriptional pattern is consistent with this requirement: by inducing *TPP*, the plant could dephosphorylate T6P and thereby uncouple it from the accumulating sucrose pool, which would be predicted to sustain SnRK1 activity and prevent sucrose from being diverted into growth. The trehalose so generated is not stored, but is either hydrolyzed (*TREHALASE* induction) or held at signaling-level amounts. Consistent with this, trehalose is only a minor constituent of the *Haberlea* sugar pool—about 0.6% of the soluble sugars in hydrated plants and about 0.2% in desiccated plants (GC–MS data) [34], far too low to constitute a dedicated protective solute. The free trehalose detected here is therefore better regarded as a by-product of T6P turnover than as a vitrifying agent. In *Haberlea*, the protective, matrix-forming role that trehalose plays in *Selaginella* is thus most likely fulfilled by sucrose and the RFOs. As T6P levels and SnRK1 activity were not measured, the proposed signaling role of the trehalose pathway remains a hypothesis consistent with the transcript pattern.

### 2.3. Relative Protective Sugar Ratio

**Figure 4:**
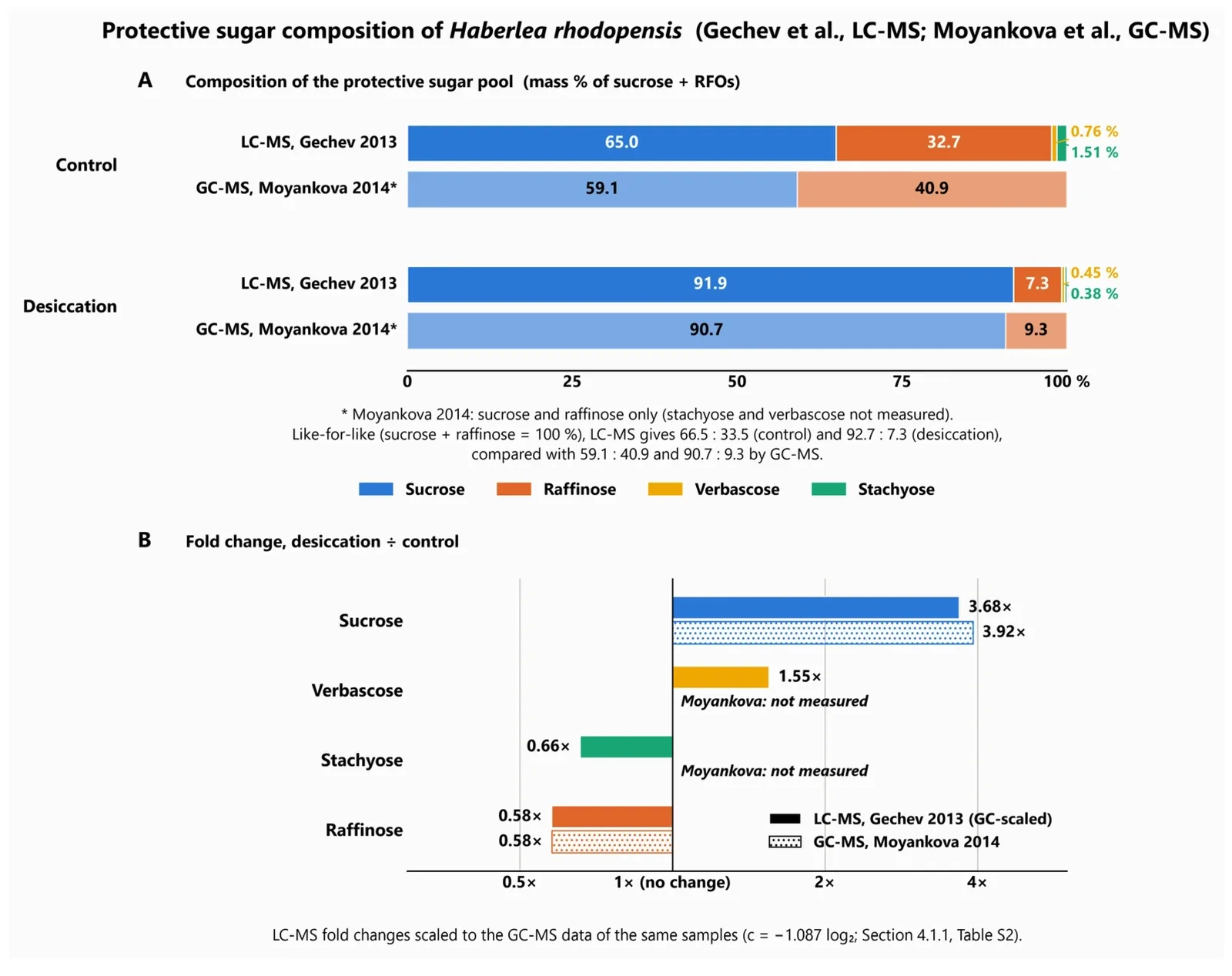
Protective sugar composition of *Haberlea rhodopensis* (Gechev 2013 [34], LC–MS; Moyankova 2014 [36], GC–MS). (**A**) Composition of the protective sugar pool (mass % of sucrose + RFOs) in the hydrated (control) and desiccated states: LC–MS, four sugars (sucrose, raffinose, verbascose, stachyose; sucrose rises from 65.0% to 91.9% of the pool), and GC–MS of Moyankova et al. [36], sucrose and raffinose only. (**B**) Change of each sugar between control and desiccation, on a logarithmic axis: LC–MS fold changes rescaled to the GC–MS data of the same samples (c = −1.087 log_2_; Section 4.1.1) and GC–MS fold changes of Moyankova et al. [36]; sucrose increases (3.68- and 3.92-fold) and raffinose declines (0.58-fold in both), whereas verbascose (1.55-fold) and stachyose (0.66-fold) were measured by LC–MS only. The GC–MS data of Gechev et al. used for the rescaling are shown in Figure 3; the LC–MS amounts and the rescaling are given in Table S2.

The analyses presented above converge on the selection of the sugars responsible for vitrification in *H. rhodopensis*. Integrating the transcriptomic and metabolomic evidence across the combined studies, four sugars emerge as the constituents of the protective glass in *Haberlea*: sucrose, raffinose, stachyose and verbascose. The protective sugar composition in the hydrated and desiccated states (LC–MS) and the change in each sugar (GC-scaled LC–MS) are shown in Figure 4. The shift towards sucrose reflects its accumulation (3.7-fold by GC-scaled LC–MS, 2.4-fold by GC–MS) together with a decline of raffinose (0.58- and 0.24-fold, respectively); an independent GC–MS dataset [36] gives closely similar changes (sucrose 3.9-fold, raffinose 0.58-fold) and a similar sucrose:raffinose composition (59.1:40.9 in hydrated and 90.7:9.3 in desiccated tissue compared with 66.5:33.5 and 92.7:7.3 by LC–MS) (Figure 4; Table S2). Notably, these four constitute a single homologous galactosyl-sucrose series consisting of sucrose and its successive α-galactosyl extensions. Such mixtures are expected to favor glass formation and hinder crystallization; for the two main components, this has been shown, as raffinose arrests sucrose crystallization and sucrose–raffinose combinations stabilize the glasses formed during desiccation [16].

*Haberlea* maintains substantial pools of sucrose and raffinose even in the fully hydrated state. Upon drying, sucrose accumulates strongly and verbascose modestly, whereas raffinose and stachyose decline. Notably, despite the pronounced transcriptional induction of raffinose synthesis, the raffinose pool is far outstripped by sucrose, which reaches an excess of approximately 13:1 at desiccation.

The role of RFOs in this context remains debated. Some studies support a contribution of oligosaccharides to seed longevity, whereas others find no direct effect. In beech (*Fagus sylvatica*) seeds, the acquisition of desiccation tolerance is closely linked to a rise in raffinose and stachyose and a marked decrease in the sucrose-to-RFO ratio, with higher oligosaccharide content associated with greater long-term storage viability, implicating these sugars in preserving seed integrity during dry storage [51]. Consistent with a protective role, a comparative survey reported that seeds with a sucrose-to-oligosaccharide ratio below one retain viability beyond ten years, whereas those with a ratio above one store for less than a decade [52]. In maize seeds, where sucrose predominates, elevated raffinose was shown to reinforce the intracellular glass, an effect associated with greater longevity [53]. Nonetheless, the relationship is not universal: in *Impatiens* and bell pepper seeds, altering oligosaccharide content had no significant effect on the stability or T_g_ of the intracellular glass [27], indicating that the role of these sugars varies among species.

The sugar “recipe” of *Haberlea*, as of any resurrection plant, is therefore likely tuned to its native habitat and evolutionary history.

### 2.4. Vitrification Strategy of H. rhodopensis

The classical route to an amorphous sugar is the one used in confectionery (hard-boiled sweets), which is one reason it was chosen here as an illustrative example. Vitrification of amorphous sugars requires the sugar to be dissolved in water and then heated to approximately 149 °C [54] into a molten state; as the water evaporates the T_g_ of the residual mixture rises, and on subsequent cooling the concentrated melt vitrifies into a disordered, amorphous configuration rather than crystallizing. Here vitrification is reached by lowering the temperature below T_g_—a vertical trajectory on the state diagram.

#### 2.4.1. Drying and Vitrification

*Haberlea rhodopensis*, like other resurrection plants, plainly does not build its glassy matrix by heating to extreme temperatures; it reaches the same physical state by an alternative strategy—the removal of water [55]. In the hydrated state, the plant’s sugars exist as a disordered solution within a mixture of biomolecules. During drying, additional sugars and protective biomolecules are synthesized, while progressive water loss concentrates them until the composition required for vitrification is reached. In this way T_g_ is raised at a fixed, ambient temperature until it exceeds that temperature—a horizontal trajectory on the state diagram.

**Figure 5:**
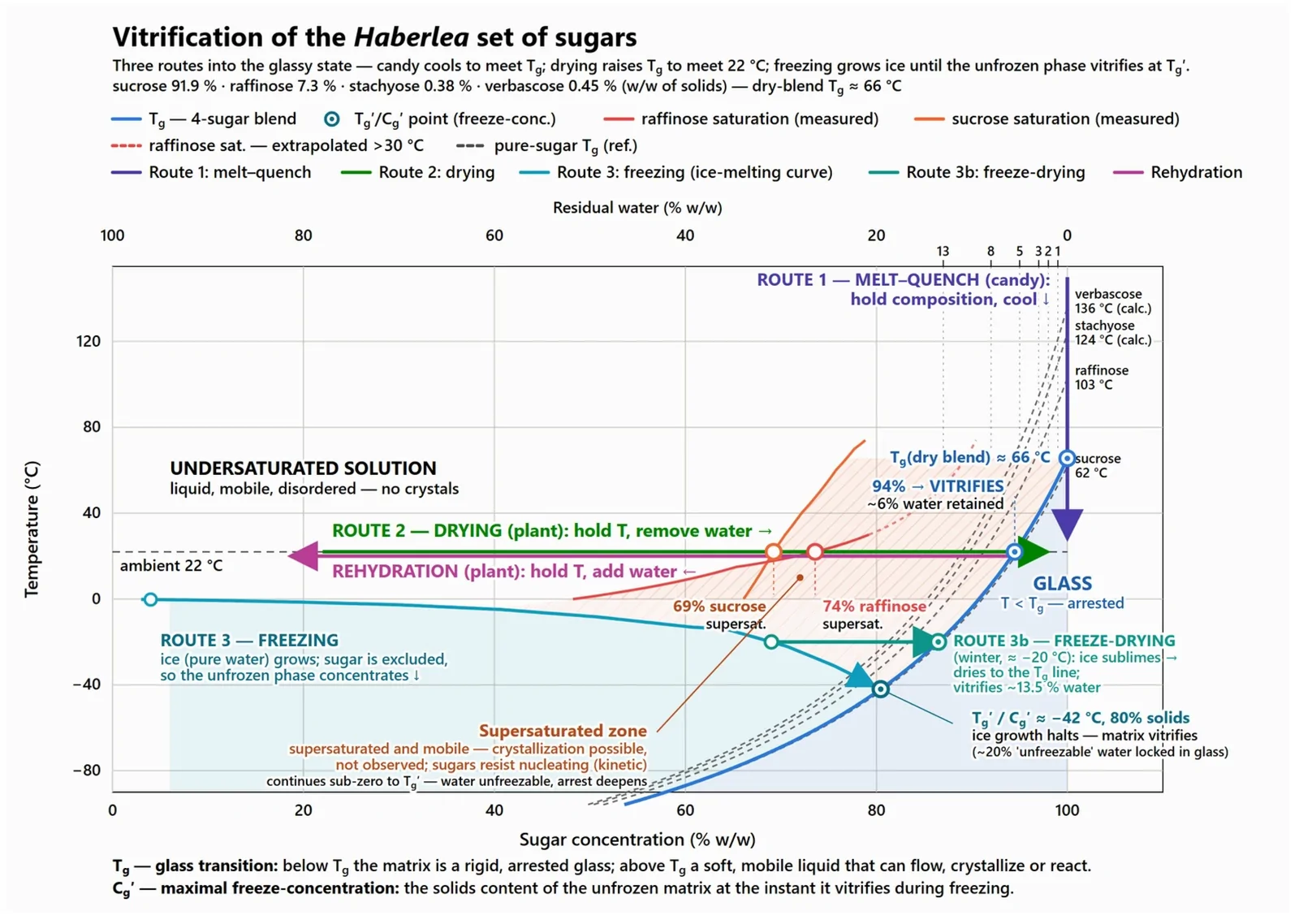
Supplemented state (vitrification) diagram of the *Haberlea rhodopensis* four-sugar blend (sucrose 91.9%, raffinose 7.3%, stachyose 0.38%, verbascose 0.45% *w*/*w* of solids). Shown are the T_g_ curve, the ice-melting (liquidus) curve, the sucrose and raffinose saturation curves, and the predicted maximally freeze-concentrated glass point, with its glass-transition temperature (T_g_′ ≈ −42 °C) and solids concentration (C_g_′ ≈ 80%; ~20% unfreezable water), together with the three routes into the glassy state—melt–quench (Route 1), drying (Route 2) and freezing/freeze-drying (Route 3/3b). Shading: light blue, glassy region (T < T_g_); light teal, ice coexisting with the freeze-concentrated solution; hatched, supersaturated zone; line colours, symbols and arrows are explained in the key above the diagram. Stachyose and verbascose T_g_ and k values are modeled (“calc.”). The diagram models the four-sugar–water system only; other cytoplasmic solutes are not included. The shaded supersaturated zone is modeled: it marks where sugar crystallization is thermodynamically possible, not where it was observed.

Both strategies are illustrated in Figure 5, where the desiccation condition is set at a representative 22 °C, which is a standard laboratory temperature at which *Haberlea* is grown and studied. It should be emphasized that the diagram is a theoretical idealization. It treats the matrix as a four-sugar–water system whose mass fractions are estimated from chromatographic data, whereas the real cytoplasm also contains other solutes, including organic acids, amino acids and proteins such as the LEA proteins, which can shift the T_g_ and viscosity of the glass. The diagram therefore describes the sugar framework of the glass and the direction in which it changes, rather than exact intracellular values. The numerical values derived from it, such as the dry-blend T_g_ (≈66 °C), the glass transition of the maximally freeze-concentrated solution (T_g_′ ≈ −42 °C) and the unfreezable water fraction (≈20%), are therefore model predictions; their direct calorimetric confirmation in *Haberlea* tissue remains to be conducted.

During desiccation (Route 2), the accumulating sugars carry an attendant risk—their nucleation and crystallization which is represented as the supersaturated zone on the diagram.

#### 2.4.2. The Supersaturated Zone

As the cytoplasm dries past the solubility limit of a given sugar, the solution becomes supersaturated while remaining soft and mobile (above T_g_). Under these conditions, the sugars are thermodynamically driven toward crystallization, since the ordered crystal is in a lower-energy state than the crowded, metastable syrup. Crystallization is not instantaneous; however, it requires the molecules first to nucleate and then to diffuse into a growing lattice, which demands both time and mobility. Herein lies the trap of the supersaturated zone—were the plant to dry too slowly, it would dwell in this supersaturated, yet mobile window long enough for nuclei to form, and the sugars would crystallize, destroying the protective glass and disrupting the membranes. *Haberlea* avoids this in two ways. First, it dries relatively rapidly [37], so the matrix stiffens and passes through the window before nuclei can organize. Second, and more importantly, it employs a mixture of sugars rather than a single one: a single, pure sugar such as sucrose crystallizes readily, whereas sucrose together with the RFOs (raffinose, stachyose and especially verbascose) differ in size and shape and cannot pack into a common crystal lattice. Interspersed, they obstruct nucleation and remain in a disordered, amorphous arrangement, while the higher viscosity contributed by the RFOs further suppresses nucleation. As water continues to leave the system, the rising T_g_ freezes this disorder into a glass. The system therefore vitrifies rather than crystallizes: crystallization is thermodynamically favored, but kinetically too slow [56,57,58,59].

#### 2.4.3. Rehydration

Rehydration follows the reverse of vitrification. Water acts as a plasticizer, lowering T_g_ steeply even for small increases in relative water content (RWC) [60]. Importantly, according to the model, on rehydration the plant retraces the same path through the supersaturated zone: as the glass softens, it briefly re-enters the supersaturated, mobile window before dilution is complete. As during dehydration, crystal nucleation [61] is inhibited there not only by the diversity of sugars—raffinose and stachyose are among the most effective inhibitors of sucrose crystallization—but also by other cytoplasmic components: even small amounts of protein inhibit the crystallization of sugar glasses [62].

Beyond its capacity to vitrify during desiccation, driven by elevated temperature and water loss, *Haberlea* also reaches this desiccated state through freeze-induced dehydration, at temperatures which can reach approximately −20 °C [63].

#### 2.4.4. Vitrification on Freezing (Route 3)

When a freezing-tolerant plant is cooled slowly below 0 °C, ice does not form within the cells, but in the dilute solution of the cell walls and intercellular spaces, where it nucleates first. Because ice has a far lower water potential than the still-liquid cytoplasm (about −1.16 MPa for every degree below the freezing point), it withdraws water from the protoplast across the plasma membrane to feed the growing extracellular crystals. The cell is thereby dehydrated from the inside out—functionally equivalent to drought desiccation (cf. Route 2), except that water is removed by ice rather than by evaporation. As water leaves, the remaining cytoplasm concentrates and the sugars accumulated within (sucrose and the RFOs) progressively raise its T_g_. The temperature continues to fall as the local T_g_ rises, and were cooling to proceed far enough the two would converge, vitrifying the maximally freeze-concentrated cytoplasm into a stable sugar glass at T_g_′ and the corresponding solids concentration, C_g_′. Once vitrified, the matrix is too viscous for water to diffuse to the ice front; ice growth halts, and the small fraction of water that never freezes remains either bound to macromolecules or kinetically trapped in the glass—leaving the cell held in a protected, vitrified state until it thaws and rehydrates [64,65,66].

#### 2.4.5. Winter Vitrification of Haberlea

Winter temperatures in *Haberlea*’s habitat remain far above the T_g_′ of about −42 °C, at which the freeze-concentrated matrix would vitrify; even at −20 °C, the model predicts that freezing alone would concentrate the sugar–water matrix only to roughly 69% solids—still a liquid, so ice by itself would not vitrify the plant. Instead, winter desiccates it: extracellular ice forms and withdraws water from the cells (freeze dehydration), and this water is subsequently lost from the tissue, most likely in part by sublimation (a natural freeze-drying), since cold air holds very little water vapor, while the frozen ground prevents the roots from replacing it. Under natural ex situ winter conditions, *Haberlea* plants indeed reach an air-dry state, with leaf RWC falling to about 12% in December and 10% in January [63]. Water is therefore not merely converted to ice, but genuinely removed from the tissue, lowering the total water content far beyond the cryoconcentration limit. In the model, freeze-drying at −20 °C reaches the T_g_ curve at a residual water content of ≈ 13.5% (*w*/*w*), at which the accumulated sugars raise T_g_ above the ambient temperature and the cytoplasm vitrifies into a stable sugar glass (Route 3b). This value is a model prediction for the sugar–water matrix and is not directly comparable to the leaf RWC values above. This is the same endpoint reached by ordinary drying (Route 2), except that here ice formation and the subsequent loss of water are the means of reaching it.

*Haberlea* is therefore by nature a poikilohydric, desiccation-tolerant resurrection plant whose freezing tolerance is an expression of that desiccation tolerance rather than a dedicated, freeze-specific adaptation: it survives sub-zero temperatures by freeze-induced dehydration, deploying the same protective machinery—sucrose, the RFOs and LEA proteins—as in warm-season drying (Route 2), consistent with the shared responses of *Haberlea* to low temperature and desiccation, which include the accumulation of sugars and of dehydrins [63]. The two situations therefore do not compete; they converge on the same outcome—the removal of water, differing only in that freezing interposes an intermediate ice step.

### 2.5. Freezing Tolerance of the Desiccated Glassy State

The *Haberlea* glass is a four-sugar system. Its maximally freeze-concentrated endpoint is set by its two abundant components—sucrose (91.9%) and raffinose (7.3%) [34], whose glass-transition parameters are derived from calorimetric measurements (Section 4.1.3). In the model, the independently parameterized glass-transition and ice-liquidus curves intersect at C_g_′ ≈ 80% solids (≈20% unfreezable water) and T_g_′ ≈ −42 °C, consistent with Roos’s calorimetric ~80% solids regularity for sucrose [66] and with raffinose’s higher T_g_ raising T_g_′ slightly above that of pure sucrose. Because these two sugars, whose parameters derive from calorimetric data, make up 99.2% of the solids, the predicted endpoint rests on experimentally based parameters and is essentially unaffected by the modeled parameters of stachyose and verbascose (<1% combined). Wherever the matrix vitrifies along the T_g_ curve it enters the same glassy state, and the residual water locked within it is unfreezable regardless of the vitrification point; only the amount of that water differs, set by the temperature at which vitrification is reached and therefore by the prevailing environmental conditions. Because this residual water cannot crystallize, deeply desiccated tissue should tolerate exposure to extreme sub-zero temperatures without the lethal intracellular ice formation that kills hydrated cells. This behavior is long established for orthodox seeds [23] and is exploited in seed cryopreservation [67]. The functional consequence of the desiccated glassy state was tested by exposing air-dry resurrection-plant material to sub-zero and cryogenic temperatures in three independent experiments.

We first tested this prediction directly in the resurrection plant *Haberlea rhodopensis*. Desiccated plants (≈10% RWC) were exposed to −80 °C for three days or immersed in liquid nitrogen (−196 °C) for 8 min, alongside a desiccated control held at room temperature, and were rehydrated 24 h after treatment and scored 24 h after rehydration. Desiccated *Haberlea* survived both freezing treatments—5/6 after −80 °C and 4/4 after liquid nitrogen—as fully as the desiccated control (4/4), whereas hydrated plants frozen identically (*n* = 4 at each of −80 °C and −196 °C) were uniformly killed (0/8; Table S3). On visual inspection, the surviving plants nevertheless showed some tissue damage after −80 °C and more pronounced damage after liquid nitrogen compared with the desiccated controls (Figure S1). Survival was significantly associated with the desiccated state at both temperatures—5/6 versus 0/4 at −80 °C (two-tailed Fisher’s exact test, *p* = 0.048) and 4/4 versus 0/4 at −196 °C (*p* = 0.029)—and more strongly when the two *Haberlea* freezing treatments were pooled (9/10 versus 0/8, *p* = 0.0004). Survival was therefore conferred specifically by the desiccated state, in which the cytoplasm is predicted to be vitrified, rather than by any intrinsic freezing tolerance of the tissue: the same plant that withstands immersion in liquid nitrogen when dry is killed by the same freezing when hydrated, because its abundant cellular water is then free to form lethal intracellular ice. Owing to the small groups, the Wilson 95% confidence intervals of these proportions are wide: 44–97% (5/6) and 51–100% (4/4) for the desiccated plants, and 0–49% (0/4) for the hydrated plants at each temperature. With four to six plants per group, the per-treatment tests are sensitive to the outcome of a single plant (one survivor fewer would raise *p* to 0.08 and 0.14, respectively), whereas the pooled comparison is not (*p* ≤ 0.003 had any one plant responded differently). Because freezing to these temperatures killed every hydrated plant, the outcome is categorical rather than a small quantitative difference; the data are therefore a proof of principle that desiccated *Haberlea* can survive such freezing, not a quantitative estimate of its survival rate.

**Figure 6:**
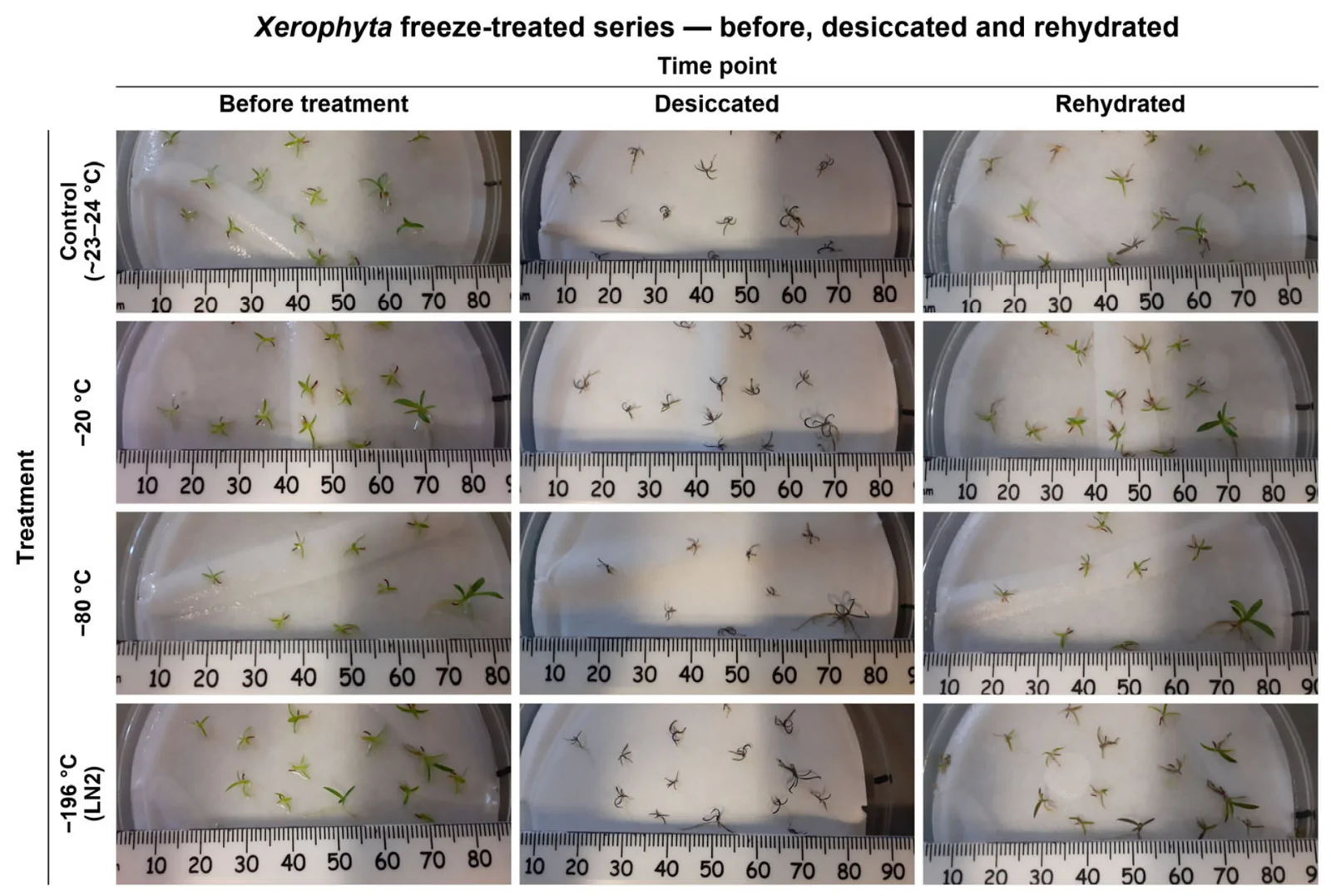
Freeze-treated series of desiccated *Xerophyta elegans* seedlings. Representative seedlings before treatment, after seven days of desiccation, and after rehydration, for the control (~23–24 °C), −20 °C, −80 °C and −196 °C (liquid nitrogen) treatments. Desiccated seedlings survived and resumed growth at all temperatures tested, including liquid nitrogen; those frozen to −196 °C showed slightly greater visible damage, but the same survival rate (*n* = 60 per condition).

We next asked whether the same behavior extends to other resurrection species, and tested desiccated seedlings of *Xerophyta elegans*, as an independent test of the principle in a second species rather than as support for the *Haberlea* result. Seedlings were slow-dried for seven days and then held at −20 °C or −80 °C for 72 h, or immersed in liquid nitrogen (−196 °C) for 1 h, alongside an unfrozen control kept at 23–24 °C; survival was scored seven days after rehydration (*n* = 60 seedlings in three dishes per condition). Survival was uniformly high and did not decline with freezing: 93.3% (56/60) in the control, 96.7% (58/60) at −20 °C, 98.3% (59/60) at −80 °C and 95.0% (57/60) after liquid-nitrogen treatment. Across the four conditions, survival did not differ significantly (Fisher–Freeman–Halton exact test, *p* = 0.71), and the Wilson 95% confidence intervals overlapped throughout (all lower bounds ≥ 84%). Freezing to −196 °C therefore caused no loss of viability relative to the unfrozen control. Seedlings exposed to liquid nitrogen showed somewhat greater visible tissue damage, but recovered at the same rate (Figure 6); per-condition survival counts are given in Table S3.

**Figure 7:**
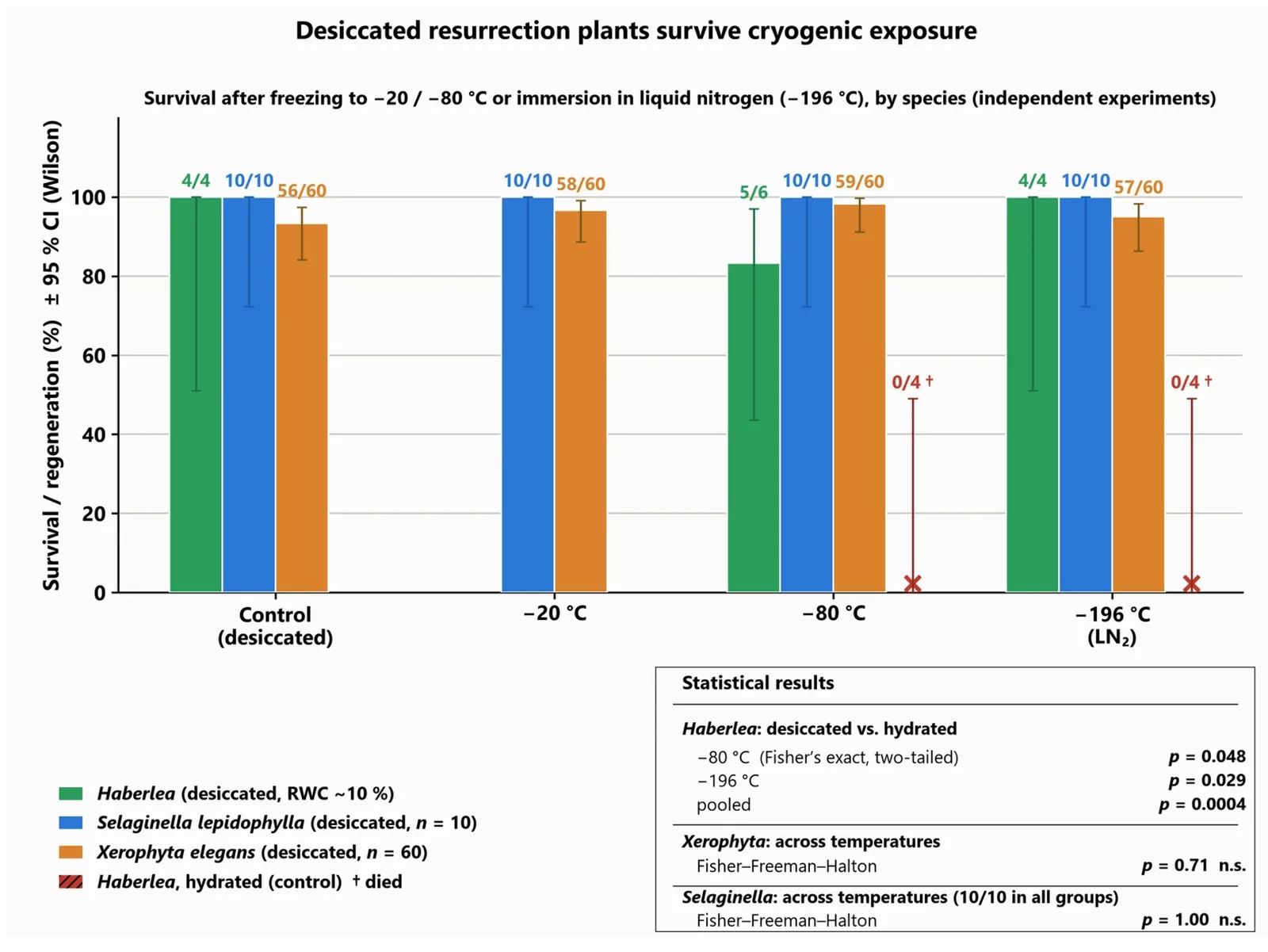
Desiccated resurrection plants survive freezing and cryogenic exposure, whereas hydrated tissue does not. Survival or regeneration (%) of three desiccated resurrection species after exposure to −20 °C, −80 °C or liquid nitrogen (−196 °C), each tested in an independent experiment: *Haberlea rhodopensis* (≈10% RWC; *n* = 4–6 per condition), *Selaginella lepidophylla* (*n* = 10 per condition) and *Xerophyta elegans* (*n* = 60 per condition). Bars show survivors/total with Wilson 95% confidence intervals. Hydrated *Haberlea* controls (red ×; *n* = 4 per treatment) were killed in both freezing treatments. Statistical tests (panel): *Haberlea* desiccated-versus-hydrated survival by two-tailed Fisher’s exact test (per treatment and pooled over the two *Haberlea* treatments); *Xerophyta* and *Selaginella* survival across temperatures by the Fisher–Freeman–Halton exact test. *Haberlea* was held at −80 °C for three days, *Selaginella* at −20 °C and −80 °C for 48 h.

**Figure 8:**
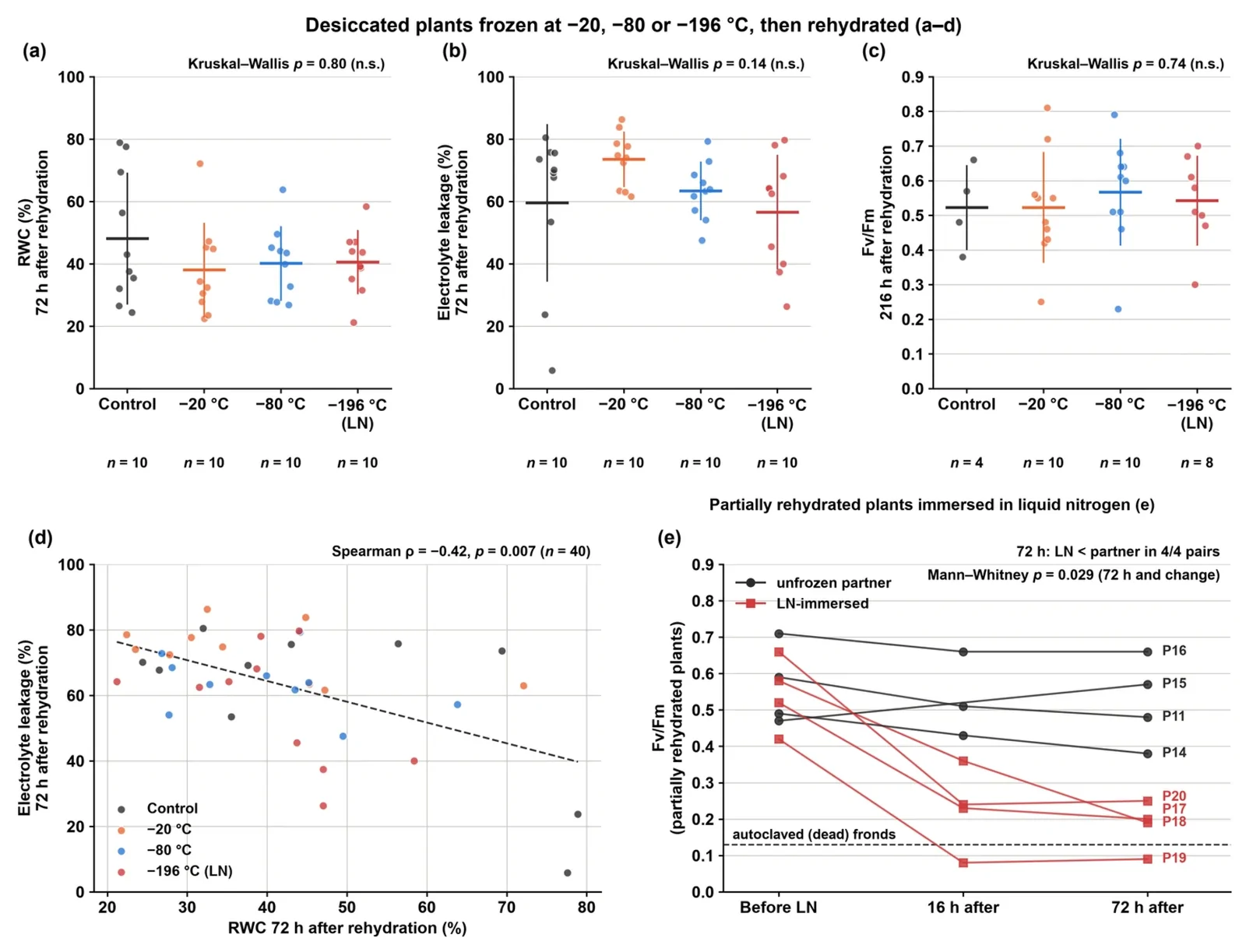
Desiccated *Selaginella lepidophylla* tolerates freezing without damage, whereas partially rehydrated plants do not. (**a**–**d**) Plants frozen in the air-dry state (−20 °C or −80 °C for 48 h, or liquid nitrogen) or kept unfrozen and then rehydrated: (**a**) relative water content (RWC) and (**b**) electrolyte leakage 72 h after rehydration, and (**c**) maximum quantum yield of photosystem II (Fv/Fm) 216 h after rehydration; dots, individual plants; horizontal bars, means; vertical lines, ±standard deviation (SD); group differences were tested with the Kruskal–Wallis test. In (**c**), the control comprises only the four unfrozen control plants (P11, P14–P16); the four control plants later immersed in liquid nitrogen as the partially rehydrated reference (P17–P20) are shown in (e), and two control plants lost to fungal infection, as well as two plants of the liquid-nitrogen group used in a preliminary test, are excluded. (**d**) Electrolyte leakage versus RWC of all 40 plants (Spearman rank correlation; dashed line, linear fit for orientation). (**e**) Fv/Fm of partially rehydrated plants (RWC 23–52%) before and 16 h and 72 h after immersion in liquid nitrogen (red squares), each paired with an unfrozen partner (black dots); lines connect measurements of the same plant; dashed line, Fv/Fm of fronds killed by autoclaving; exact two-sided Mann–Whitney U test. The 16 h value of P15 is omitted (capture artifact). *p* values are given in the panels; data in Table S4.

The same held in a resurrection species of independent evolutionary origin, the lycophyte *Selaginella lepidophylla*. Air-dry plants (RWC 11.5 ± 2.3%; *n* = 10 per condition) were held at −20 °C or −80 °C for 48 h or immersed in liquid nitrogen, alongside an unfrozen control, and then rehydrated. All plants survived: they unrolled and turned green within 24–48 h of rehydration, irrespective of treatment (Figure 7 and Figure S2a). At 216 h after rehydration, the maximum quantum yield of photosystem II (Fv/Fm) did not differ among the four groups: 0.52 ± 0.12 in the control, 0.52 ± 0.16 at −20 °C, 0.57 ± 0.15 at −80 °C and 0.54 ± 0.13 after liquid nitrogen (Kruskal–Wallis test, *p* = 0.74; Figure 8c; Table S4). The four groups also regained water to the same extent: 72 h after rehydration, their RWC did not differ (38–48% on average; Kruskal–Wallis test, *p* = 0.80; Figure 8a), so freezing, even in liquid nitrogen, did not impair the capacity of the plants to take up water again; at this time all groups were still only partially rehydrated. Electrolyte leakage, measured at the same time, did not differ among the groups either (*p* = 0.14; Figure 8b). Instead, it varied with the degree of rehydration of the individual plants: across all 40 plants, regardless of treatment, leakage was lower the more water a plant had regained (Spearman ρ = −0.42, *p* = 0.007; Figure 8d). The differences in leakage between plants thus reflected how far each plant had rehydrated, not the temperature to which it had been exposed. One plant in each frozen group had a low Fv/Fm (0.23–0.30), which did not alter these comparisons.

As a positive control, eight control plants were used six days after rehydration, when they were still only partially rehydrated (RWC 23–80%). They were matched in pairs by their electrolyte leakage 72 h after rehydration and by visual comparison, and one plant of each pair was immersed in liquid nitrogen in the same way. In the four frozen plants (RWC 23–52% at freezing), Fv/Fm fell from 0.55 ± 0.10 (0.42–0.66) before freezing to 0.23 ± 0.11 at 16 h and 0.18 ± 0.07 (0.09–0.25) at 72 h, close to that of fronds killed by autoclaving (0.13), indicating that photosystem II had largely lost its photochemical activity; their unfrozen partners remained at 0.56 ± 0.11 before freezing, 0.53 ± 0.12 at 16 h (*n* = 3; one reading excluded as a capture artifact) and 0.52 ± 0.12 at 72 h. Every frozen plant ended below its partner (exact Mann–Whitney test, *p* = 0.029 at 72 h and for the change from before freezing; Figure 8e). Immediately after the treatment the frozen plants still looked green, only slightly darker than the unfrozen plants, but over the following days they turned brown (Figure S2c). Fv/Fm of the partially rehydrated plants after liquid nitrogen was also lower than that of the desiccated plants given the same treatment (*p* = 0.004). With four plants per group this reference is qualitative, but it shows that the liquid-nitrogen treatment that left desiccated *Selaginella* undamaged severely damaged the same plants when they held more water. This lycophyte, evolutionarily far removed from *Haberlea* and *Xerophyta*, thus also survives freezing, including immersion in liquid nitrogen, in the desiccated state. Unlike desiccated *Haberlea* and *Xerophyta*, which survived liquid nitrogen, but showed visible tissue damage, most pronounced in *Haberlea*, desiccated *Selaginella* showed no more damage than the unfrozen controls by any of the measures used.

Together, these results (Figure 7 and Figure 8) provide strong functional support for the presence of a protective glassy matrix in resurrection plants and for its parallel with the glassy matrix of orthodox seeds.

The purpose of the glassy matrix is to “freeze” and halt the plant’s cellular processes, and spin-probe EPR confirms this near-complete arrest of molecular mobility [26]. What, then, happens when desiccated *Haberlea* is exposed to ultralow temperatures? Thermodynamically, almost nothing. The matrix is already below T_g_, which implies it is solid, amorphous and kinetically arrested, so cooling it to −196 °C only makes it more kinetically stable—the system becomes a colder, more rigid version of itself [25]. Its water content is too low to contain the water clusters (larger than ~90 molecules) required to nucleate crystalline ice [68]; the little water that remains is bound and cannot migrate to a growing crystal [69]; and the sugars are kinetically locked and unable to reorganize. The only real hazard at such temperatures is mechanical rather than thermodynamic: the abrupt contraction of rapid cooling can impose thermomechanical (fracture) stress on the brittle glass [70], which may account for the tissue damage observed after freezing in some species—pronounced in desiccated *Haberlea*, slight in *Xerophyta* and not detected in *Selaginella*—without compromising the glassy state itself.

**Figure 9:**
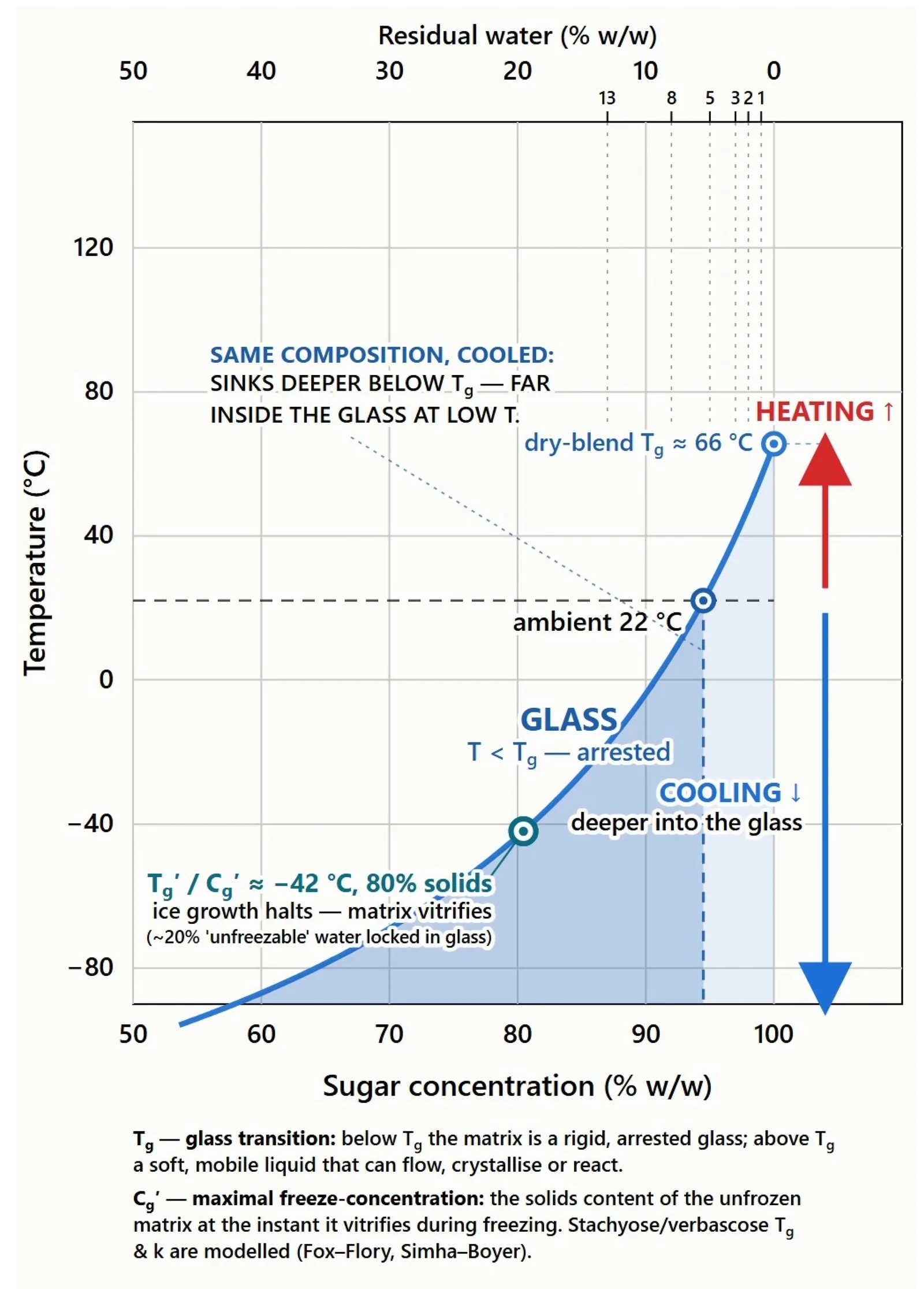
Modeled effect of temperature on the vitrified four-sugar glass of *Haberlea rhodopensis*. At the ambient desiccation point (22 °C, ≈94% solids/≈ 6% residual water) the composition lies on its glass-transition curve (T_g_ = 22 °C at this water content; dry-blend T_g_ ≈ 66 °C); cooling drives the same composition deeper into the arrested glassy state (T < T_g_), while heating moves it toward the mobile liquid. Light blue, glassy region (T < T_g_); darker blue, the margin by which the cooled desiccated composition (vertical dashed line, ≈ 94 % solids) lies below its T_g_; red and blue arrows, heating and cooling of this composition. The maximally freeze-concentrated point (T_g_′/C_g_′ ≈ −42 °C, ≈80% solids) marks where ice growth halts and ≈20% unfreezable water is locked in the glass. Top axis, residual water (% *w*/*w*); stachyose and verbascose T_g_ and k are modeled (Fox–Flory, Simha–Boyer).

The effect of lowering the temperature is illustrated in Figure 9. Below the vitrification point (22 °C), the matrix lies far beneath its glass transition (T ≪ T_g_), its solids content greatly exceeding the minimum concentration required to vitrify at that temperature. The further below T_g_ the system sits, the longer its structural (α) relaxation time; accordingly, the wider the interval (dark blue) between the T_g_ curve and the actual composition, the greater the kinetic stability of the glass. Warming works in the opposite direction: as the system approaches T_g_ the relaxation time shortens, reducing the longevity of the matrix, and a still-lower water content would be required to keep T_g_ above the ambient temperature. In theory, held under such conditions, desiccated *Haberlea* could remain viable for decades to centuries [19,57]—precisely the principle exploited in seed cryopreservation [67].

### 2.6. Thermodynamics of the Glassy Matrix

The glass transition is a kinetic rather than a thermodynamic phase transition: T_g_ is rate-dependent, shifting upward under faster cooling or drying, and it involves no latent heat—only a step increase in C_p_ on passing from glass to liquid. Glass and liquid are therefore not in equilibrium at T_g_; rather, at T_g_ the system falls out of equilibrium, because its molecules can no longer rearrange quickly enough to track the changing conditions and become frozen in whatever configuration they occupied [71]. As water leaves the system, water–water and sugar–water hydrogen bonds are progressively lost while sugar–sugar bonds form in their place [72], and the protective sugars hydrogen-bond directly to proteins, membranes and other biomolecules—the water-replacement hypothesis [49,73]. The water that remains is held more strongly than in liquid water [72], and the dried glass itself is hygroscopic [73]. In a trehalose glass, density-functional calculations place the binding energy of the individual residual water molecules at roughly 52 to nearly 100 kJ/mol, the exact value depending on each molecule’s local environment; and because the glass relaxes far too slowly for its constituent molecules to reorganize and compensate for the loss or gain of water, hydrogen-bond substitution is incomplete and vacant binding sites persist—removing one water molecule restores only ~0.73 trehalose–trehalose hydrogen bonds [72]. For the same reason, rehydrating a dry amorphous sugar is markedly exothermic: the incoming water occupies these pre-existing vacancies; each added molecule forms about three hydrogen bonds with the sugar while breaking only ~0.73 sugar–sugar bonds [72]. Compared with water in the pure liquid, this amounts to a net gain of ~0.7 hydrogen bonds per molecule, released as heat on the order of −18 kJ/mol [72,74]. This asymmetric, exothermic hydration is a hallmark of amorphous sugar glasses.

**Figure 10:**
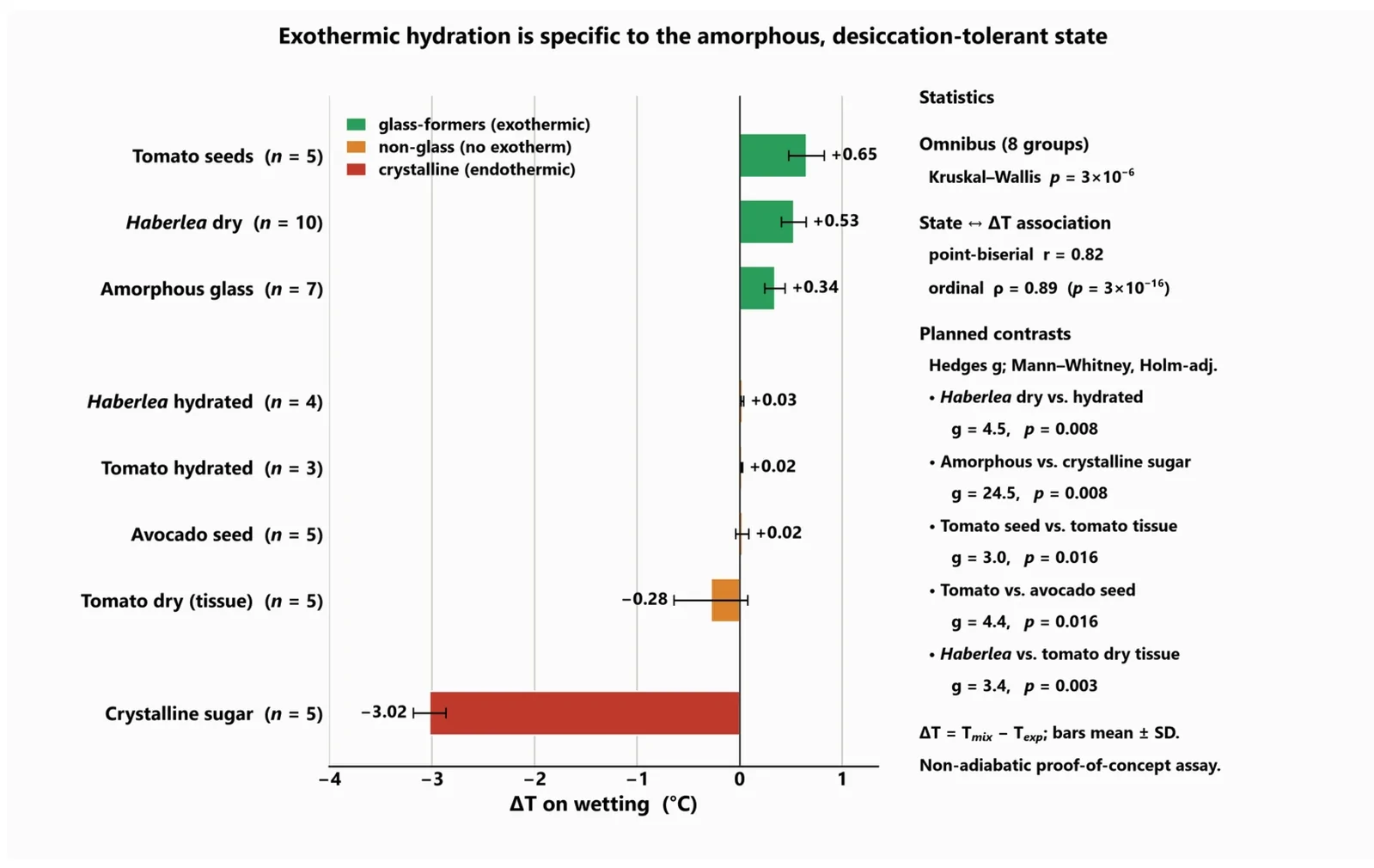
Exothermic hydration is specific to the amorphous, desiccation-tolerant state. Temperature change on wetting each material with water (ΔT, the mixture temperature minus the expected no-reaction mixing temperature), by group; bars are mean ± SD, with *n* indicated per group. Glass-forming, desiccation-tolerant materials—desiccated *Haberlea*, a melt-quenched 80:20 sucrose–glucose glass and orthodox tomato seeds released heat, whereas non-glass materials showed no exotherm and the crystalline sucrose–glucose standard was endothermic. The side panel reports the omnibus Kruskal–Wallis test, planned Mann–Whitney contrasts (Holm-adjusted) with Hedges’ g effect sizes, and the point-biserial and ordinal correlation of ΔT with physical state. The assay is a simple, non-adiabatic proof-of-concept measurement: ΔT values are comparable between groups measured under identical conditions, but are not absolute calorimetric quantities.

To test this property directly, as a simple proof-of-concept assay, we measured the heat released when desiccated materials were wetted, using the temperature rise on mixing with water as a simple heat-of-wetting measurement (Figure 10). Desiccated *Haberlea* produced a clear, reproducible exotherm (ΔT = +0.53 ± 0.12 °C; values are mean ± SD), as did the two other amorphous, glass containing materials tested—a melt-quenched 80:20 sucrose–glucose glass (+0.34 ± 0.10 °C) and orthodox tomato seeds (+0.65 ± 0.17 °C). By contrast, none of the non-glass-forming materials warmed on wetting: hydrated *Haberlea* and tomato tissue and the recalcitrant avocado seed were flat (ΔT ≈ +0.02–+0.03 °C), dried tomato vegetative tissue showed no exotherm (−0.28 ± 0.36 °C), and the crystalline sucrose–glucose standard of identical composition was strongly endothermic (−3.02 ± 0.16 °C), as expected for the dissolution of crystalline sugar. The eight groups differed overall (Kruskal–Wallis, *p* = 3 × 10^−6^), and the exotherm was specifically associated with the amorphous, desiccation-tolerant state rather than with the organism or its tissue chemistry (point-biserial r = 0.82; ordinal ρ = 0.89, *p* = 3 × 10^−16^; restricted to the biological materials, r = 0.86 and ρ = 0.87, both *p* < 10^−9^). Desiccated *Haberlea* differed sharply from its hydrated counterpart (Hedges g = 4.5) and the amorphous glass from the chemically identical crystal (a very large effect, g = 24.5); notably, orthodox tomato seeds, which vitrify, differed both from dried vegetative tissue of the same plant, which does not (g = 3.0, *p* = 0.016), and from the recalcitrant avocado seed (g = 4.4, *p* = 0.016); and, comparing dried vegetative tissues directly, the desiccation-tolerant *Haberlea* tissue released heat where desiccation-sensitive tomato tissue did not (g = 3.4, *p* = 0.003). The full matrix of pairwise effect sizes across all eight groups is provided in Figure S3. Because the assay was not adiabatic (Section 4.8), these ΔT values are not calorimetric measurements of heat release; they are interpreted only comparatively, between groups measured under identical conditions, and not as absolute thermodynamic quantities. An exotherm on rewetting is not in itself conclusive proof of a glassy state, because other processes, such as the rehydration of cell walls, the dissolution of hydrocolloids, imbibitional leakage or the restart of metabolism, can also release heat when dried tissue is wetted. These are unlikely to explain the present signal: the exotherm was absent from the dried vegetative tissue of a desiccation-sensitive plant, which contains the same cell walls and hydrocolloids and could restart its metabolism in the same way, so these processes did not produce a detectable heat release under the assay conditions, and from the starch-rich recalcitrant seed, while the crystalline standard of identical composition was endothermic; the dried materials were ground before the assay, which disrupts cellular organization; and the temperature peak was reached within one to two minutes of wetting, so the signal tracks the amorphous state rather than tissue chemistry in general. The exothermic hydration of dried *Haberlea* is therefore the thermal signature expected of an amorphous sugar glass [74], providing a second, independent line of evidence next to the freezing-survival result that its desiccated cytoplasm forms a glassy matrix.

### 2.7. Distribution and Functions of Residual Water

#### 2.7.1. Distribution

Whether the residual, non-freezing water plays a functional role in desiccated resurrection plants or is simply what remains once drying is complete is still an open question. An aquaphotomics (near-infrared) study of the plant [75] found that at full desiccation (RWC ~ 13%) the proportions of water species carrying different numbers of hydrogen bonds are strongly redistributed, a change not seen in the desiccation-sensitive relative *Deinostigma eberhardtii*. In *Haberlea*, free water (S0) collapsed from 57% to 13% of the water population at full desiccation, whereas in *D. eberhardtii* it remained near 52% at its lethal limit of drying (about 35% RWC)—a fundamental difference in how the two plants handle water loss. As free water fell, *Haberlea* reorganized the remainder into hydrogen-bonded species: at full desiccation, water with two and three hydrogen bonds (S2, S3) increased slightly, whereas the most striking changes were the rise of water with four hydrogen bonds (S4) and the massive accumulation of water dimers (S1). Kuroki et al. identify the massive accumulation of “water dimers” (the S1 species, ~1440 nm) at full desiccation as *Haberlea*’s most striking water-structure change, yet leave its origin explicitly open, unable to say whether the dimers are a by-product of the desiccation-associated biochemistry or are accumulated for a specific purpose. Their clearer structure-making argument, in which the accumulating sucrose and raffinose promote hydrogen bonding in the residual water, attaches most naturally to the rise in fully coordinated (S4) water, while the S1 dimers remain unaccounted for.

Here we offer an alternative reading of these published data rather than a new measurement. The band assignment underlying the S1 species is taken from pure water: in the supplementary analysis its position is taken from temperature-dependent spectra of pure water [76,77], and although several carbohydrate O–H bands are resolved elsewhere in the spectrum (near 1574, 2016 and 2414 nm), no sugar contribution is assigned in the S1 region. Yet a non-bonded hydroxyl band lies within a few nanometers of S1: in processed sucrose the non-bonded O–H first overtone absorbs at ~1436 nm and changes shape between the amorphous and crystalline states [78], and a later aquaphotomics study from the same group as Kuroki assigns this ~1436 nm feature to a non-bonded-OH, water–carbohydrate-interaction band tied to the phase transition of the matrix [79]. Such free hydroxyls are moreover consistent with what the glass physics predicts: because the vitrified matrix relaxes too slowly for its molecules to reorganize as water departs, sugar hydroxyls that lose their water partners are stranded as non-bonded OH rather than forming new sugar–sugar bonds—the incomplete hydrogen-bond substitution that also makes rehydration of amorphous sugars strongly exothermic [72,74]. We therefore hypothesize that the S1 “water-dimer” accumulation in dried *Haberlea* may, in part, reflect the free (non-bonded) hydroxyl signal of the vitrifying sucrose and raffinose themselves—the very sugars whose accumulation coincides with it, rather than free water–water dimers, so the correlation between S1 and sugar content may reflect spectral overlap as much as a causal link. This alternative interpretation remains to be tested directly, for example by recording near-infrared spectra of anhydrous amorphous sugar glasses of the same composition.

Even the fraction of the S1 signal that is genuinely water need not be free dimers. The aquaphotomics classification sorts water by the strength of the single hydrogen bond it forms—read from the position of its O–H stretch and not by the identity of the partner, so a water molecule bound to a biomolecular surface by a single bond of comparable strength is recorded together with a water–water dimer. At ~13% RWC, classical hydration theory makes free dimers improbable: Brunauer–Emmett–Teller (BET) adsorption leaves essentially a single surface layer [80,81], the residual water is heavily outnumbered by the biomolecular binding sites available to it, and decades of research place low-hydration water as a monolayer bound singly at polar sites [82,83]. An isolated water–water dimer, moreover, could persist only in a hydrophobic pocket, where no competing polar groups are present to bond it apart [84,85]; in the hydroxyl-rich sugar glass a water molecule would simply hydrogen-bond to the surrounding sugar and cease to be a dimer. We therefore consider it more likely that the water contribution to S1 comes from surface-bound monolayer water than from free dimers, although dimers trapped in cavities formed by the specific geometry and limited relaxation of the biological glass cannot be excluded.

Our liquid-nitrogen experiment indicates that whatever the water species, the residual water behaves as one of the bound, confined and interfacial forms long described at protein hydration shells, membrane surfaces and in nanoscale pores—water that can take on locally ordered, ice-like arrangements yet stays kinetically trapped and does not freeze into bulk ice even well below 0 °C [83,86,87]. Moreover, wherever this water is organized into clusters, those clusters hold only a handful of molecules—fewer than the ~90 that mark the minimum for ice nucleation [68]. The tissue damage observed after freezing (Section 2.5; Figure S1), beyond the fractures attributable to thermomechanical stress on abrupt cooling, most plausibly reflects a small fraction of incompletely removed water whose clusters remain large enough to cross the nucleation threshold.

#### 2.7.2. Functions

The importance of water in cell biology is easily underestimated: far from a passive solvent, its bound forms act as active, structural participants in biomolecular function. Molecular dynamic studies of protein association show that conventional thermodynamic models reproduce the experimental binding affinities only when the slow, interfacial waters are treated explicitly as part of the complex [88]. These “bridging” waters hydrogen-bond to two partners at once, filling gaps between surfaces that would not otherwise fit, while the more weakly held single- and double-hydrogen-bonded waters of the hydration shell form more stable contacts than bulk water and stabilize the biomolecular surface even in the unbound state; because both relax far more slowly than free water, they govern biomolecular dynamics and set the energetics of molecular recognition, catalysis and nucleic-acid stability [88,89]. The same holds for the residual water of dehydrated tissue: even lyophilized proteins remain 5–8% hydrated (*w*/*w*) [90], its complete removal drives unfolding, aggregation and loss of function [91], and water itself is integral to the native structure of proteins and of the DNA double helix [92]. In membranes, 10–12 water molecules are hydrogen-bonded around each phospholipid phosphate; their removal raises the lipid phase-transition temperature, so dry bilayers are prone to fusion and leak as they pass through the transition on rehydration [49].

In hygroscopic plant (cellulosic) tissues, water moves in two steps: it is first absorbed into the cell wall, and this bound water then acts as the vector that carries free water through the material [93]. Because of this wall-mediated step, imbibition in cellulose is 10–100× slower than the Lucas–Washburn capillary prediction—a regulatory, protective behavior that prevents abrupt, potentially destabilizing hydration by liquid water [94]. Consistently, free liquid water appears in the cell lumina only once the walls are saturated with bound water (the fiber-saturation point), and on drying the bound fraction begins to leave only after all local free water has gone, with dehydration below fiber saturation proceeding markedly faster than uptake [95]. Although demonstrated in wood, the authors hold these principles to be general across vascular plants. These principles could play an important role in the rehydration processes of resurrection plants.

#### 2.7.3. Function in the Glassy Matrix

When a biological glass forms in drying cells, the water content never falls to zero: a residual fraction of ~0.05–0.3 g H_2_O g^−1^ dry biomass is always retained, and it is unfreezable, not crystallizing even when cooled to −150 °C [23]. This water is confined, kinetically slowed and structurally distinct from bulk water [26], and it is an integral component of the sugar–glass matrix, where it acts as a plasticizer—a material incorporated to increase workability, flexibility or extensibility [19]. As a result, even a very small change in water content lowers the T_g_ sharply [25].

This residual water is therefore essential. Fully dry sugar glasses (~0% H_2_O) are often more susceptible to damage: they lose their protective function as proteins and membranes destabilize, and at extreme dryness local holes have been proposed to form in the glassy matrix, through which small molecules such as oxygen and free radicals could diffuse despite the glassy state [19], accelerating aging and shortening longevity [28,96]. An optimum—a “golden mean”—therefore exists for residual water content [97].

Taken together, these considerations suggest a broader role for the accumulated biomolecules. Beyond substituting for water at the surfaces it vacates (the water-replacement hypothesis), the sugars and proteins may act to preserve each macromolecule’s hydration monolayer—retaining the minimal, tightly bound water that keeps essential structures from irreversible damage, and so safeguarding the machinery on which rehydration and regeneration depend. Water replacement and retention of the hydration monolayer would then be two complementary faces of the same adaptation.

### 2.8. LEA Proteins

The four-sugar blend used in the state diagrams is a deliberate simplification, adopted to make the vitrification mechanism and the physical behavior of the matrix tractable to analysis; in the living cell, however, the cytoplasm vitrifies as a crowded mixture of many different biomolecules and other solutes, some of which materially change the biophysical properties of the glass [31]. Prominent among these are the LEA proteins, which act not only as molecular shields [98,99], but in some cases also modify the properties of the glass itself: in vitro, they raise the T_g_ of dry sucrose glasses and strengthen their hydrogen-bond network [100,101], and in *Arabidopsis* seeds the loss of a group 6 LEA protein made the glass more fragile without changing its T_g_ [102].

The critical temperature (Tc) is the temperature at which molecular motion within the glass shifts from solid-like to liquid-like. In pure sugar glasses Tc lies only 17–35 °C above the T_g_, whereas in biological glasses this interval widens to at least 50 °C, and proteins rather than sugars are thought to be responsible for this enlarged stability window, with the abundant LEA proteins as likely candidates [46]. According to the published expression data of the *Haberlea* genome study [35], an *LEA* transcript is among the most abundant of all transcripts even in the fully hydrated state, and several further *LEA* transcripts are also highly expressed. Together with its high constitutive sugar levels, this is consistent with reports that the plant is constitutively “primed” for desiccation [34].

### 2.9. Candidate NaDES-Forming Metabolites Hypothesis

NaDESs are liquids formed when a hydrogen-bond donor and acceptor, such as a sugar, polyol, organic acid or amino acid, associate in particular proportions and depress each other’s melting point, yielding a fluid phase that persists at very low water content [103]. Such solvents have been proposed to provide a localized liquid phase that supports residual metabolism in desiccation-tolerant plants below ~30% RWC, before the cytoplasm vitrifies [103,104,105], and in dehydrating *Brassica napus* seeds sucrose formed hydration-dependent, sucrose-centered assemblies with malate and several amino acids, detected in seed extracts and tissue sections and reproduced in bioinspired artificial mixtures, in which glucose and fructose formed no such assemblies, whereas raffinose and trehalose behaved like sucrose [106]. Whether a comparable phase forms in *Haberlea* has not yet been examined directly.

**Figure 11:**
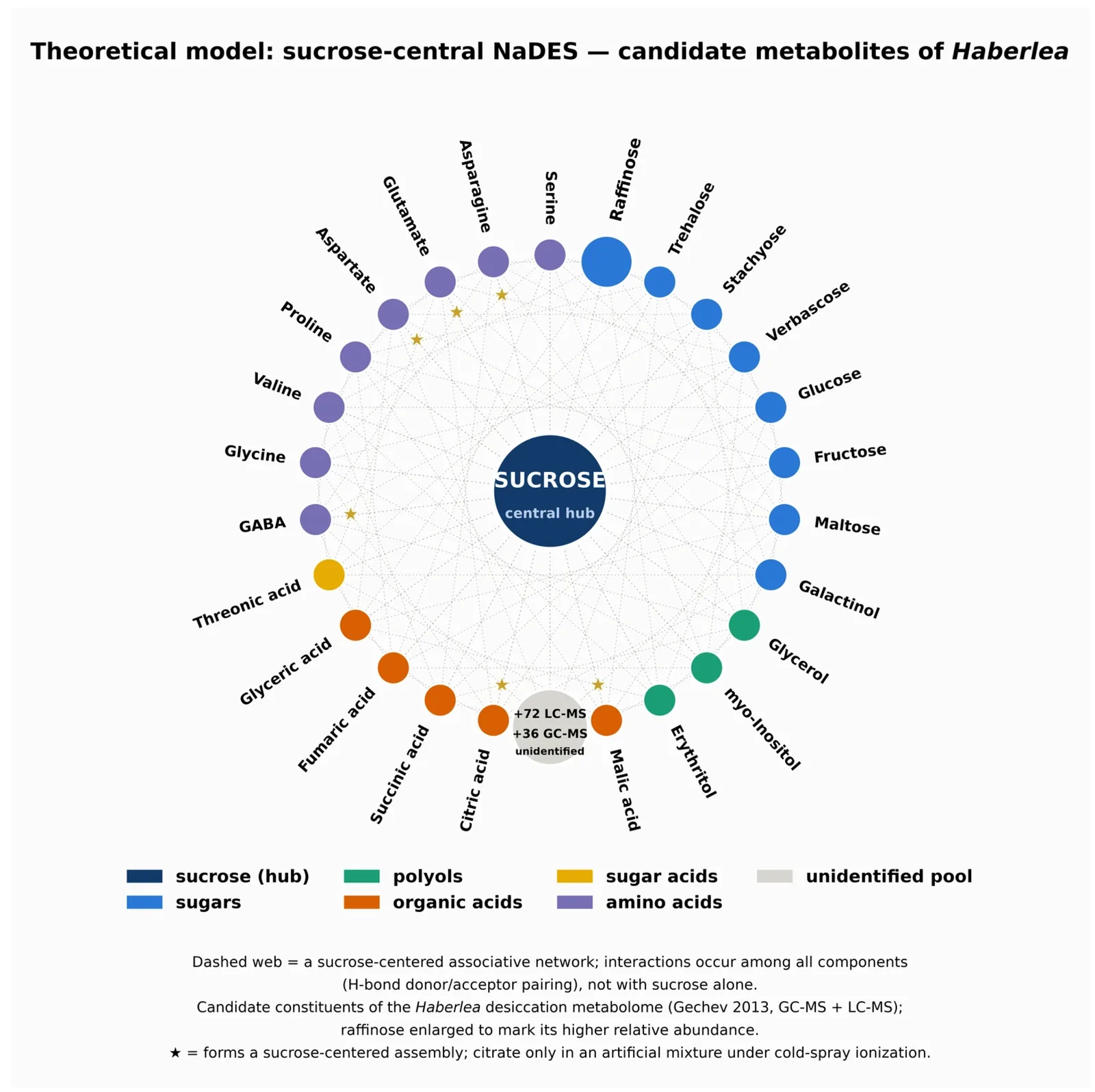
Theoretical model: candidate NaDES-forming metabolites of *Haberlea*. Sucrose (center) and identified desiccation-metabolome constituents whose hydrogen-bonding chemistry is suitable for NaDES formation [103,104] (Gechev 2013 [34], GC–MS + LC–MS), colored by class. Inclusion reflects presence and chemical suitability only; eutectic behavior has not been measured in *Haberlea*. ★ = forms a sucrose-centered assembly in *Brassica napus* seeds [106]; citrate only in an artificial mixture under cold-spray ionization.

Its desiccation metabolome, however, contains the classes of metabolites from which NaDESs are known to form and that have been proposed to act in vegetative desiccation tolerance [103,104]: sucrose accumulates strongly, γ-aminobutyric acid (GABA) increases, and malate, asparagine, glutamate and aspartate, although they decline, remain present in the desiccated tissue [34] (Figure 11). We therefore hypothesize that dehydration of *Haberlea* may drive an analogous sucrose-centered, NaDES-like assembly, extending the metabolically active window during late drying and early rehydration before the matrix vitrifies. The hypothesis makes testable predictions: it would be supported by imaging mass spectrometry detecting sucrose-centered adducts in dried *Haberlea* tissue, by a eutectic melting or glass-transition signal in differential scanning calorimetry (DSC) distinct from that of the sugar glass, and by enzymatic activity retained at low water content. Conversely, the absence of a separate liquid phase in drying tissue below ~30% RWC, that is, no additional thermal transition by DSC and no mobile liquid-like fraction by nuclear magnetic resonance (NMR) relaxometry, would refute it.

## 3. Discussion

Resurrection plants have evolved an elegant protective system in which every step of the desiccation process appears finely coordinated toward the same end. In the vitrification view of anhydrobiosis [19,57], vitrifying rather than crystallizing spares the cell both the mechanical damage that growing crystals would inflict and the energy their redissolution would require on rehydration. Once the cytoplasm vitrifies, molecular mobility is slowed to a near standstill and the cell enters a state of suspended animation: solutes, proteins and membranes are held in the positions they last occupied, and the diffusion-dependent reactions that would otherwise consume or damage them—including oxidative chemistry—are halted. This arrest is purely kinetic and fully reversible: when water returns and plasticizes the glass, mobility is restored and cellular processes resume from where they were suspended, requiring no additional energy to set them back in motion. Most strikingly, our results suggest that the very mechanism that protects the plant against desiccation also protects it against freezing, once it is dry.

What that strategy looks like in molecular detail is the subject of this work. Bringing the transcriptomic and metabolomic records of *Haberlea rhodopensis* into a single framework and reading them through the physics of the amorphous state yields a coherent model of how this plant vitrifies. The glass that forms on drying is built not from one sugar, but from a graded, sucrose-dominated family—sucrose together with its successive galactosyl extensions raffinose, stachyose and verbascose—a composition expected to resist crystallization because its members cannot share a common lattice. Two transcriptional features that at first seem to contradict this picture appear, on closer inspection, consistent with it. The strong induction of *INVERTASE* genes alongside pronounced sucrose accumulation can be explained by compartmentation: the induction is concentrated in organellar and apoplastic (cell-wall) isoforms with no access to the vacuolar sucrose store, while the one dominant vacuolar invertase is repressed. Similarly, induction of the trehalose pathway as trehalose itself falls is best explained by the signaling economy of T6P rather than by a second vitrifying sugar; both explanations rest on transcript data and remain to be tested at the protein and metabolite level. Read together, transcription and metabolite levels help to distinguish the molecules the plant makes to build its glass from those merely present within it.

A single physical idea unifies the routes into that glass. Whether water leaves by evaporation in a warm, dry summer or by sublimation of extracellular ice in a cold, dry winter, in our model the cytoplasm climbs the same glass-transition curve to the same vitrified endpoint; freezing merely interposes an intermediate ice step. This carries a consequence that is easy to miss. In still-hydrated tissue, winter cold elicits the physiological and metabolic responses of a cold-acclimating plant, and these serve to protect the living, hydrated cell against chilling and freezing injury [63]; there is little indication that the plant assembles a drying program tailored specifically to freeze-dehydration. The protection that ultimately survives the winter appears to be not a cold-specific structure, but the ordinary desiccated glass: once the cytoplasm has vitrified, the temperature and the route by which its water departed are immaterial. The survival of desiccated tissue at −196 °C strongly supports this view. In our experiments, hydrated plants were killed by freezing at such temperatures, whereas desiccated plants, whose matrix is already solid, water-depleted and kinetically arrested, survived immersion in liquid nitrogen. Survival of desiccated leaves of the closely related resurrection plant *Ramonda myconi* after immersion in liquid nitrogen was reported in an early study, and in that species desiccation tolerance has been proposed as the basis of frost resistance too (both discussed in [107]). *Haberlea* therefore appears to hold no dedicated machinery for freeze-vitrification: its freezing tolerance in the dry state is not a separate adaptation, but its desiccation tolerance seen from another angle—the same glass, reached at a lower temperature.

The analysis also reframes the water that remains in the glass. Far from an inert remainder, this residual fraction appears to be bound, kinetically slowed and structurally distinct, and the cryogenic survival of dry tissue indicates that it does not form damaging ice. In this light, the near-infrared “water-dimer” signal that rises so steeply on drying may not report free water alone: we hypothesize that part of it arises from the non-bonded hydroxyls stranded on the vitrifying sugars, and from water bound singly to biomolecular surfaces, rather than from genuine water–water pairs. The distinction matters less than the shared conclusion—the water is immobilized within the matrix. We propose that its role there is twofold and complementary: the accumulated sugars and proteins substitute for water at the surfaces it vacates, and we hypothesize they help to preserve the last, tightly held monolayer, whose loss would likely be irreversible.

Sugars, however, are only the tractable core of a more crowded reality. In the cell, the glass forms within a mixture of biomolecules, and at least two classes are expected to tune its properties. LEA proteins, whose transcripts are abundant in *Haberlea* even before drying [35], act as molecular shields and in vitro stiffen sugar glasses and raise their glass transition [100]—plausibly contributing to the unusually wide stability window of biological, as opposed to purely sugar, glasses [46]. Direct evidence that the biological glass is more than its sugars comes from the resurrection plant *Craterostigma plantagineum*, in which the dried cytoplasm showed a higher glass transition than a pure sucrose glass; the authors suggested that soluble cytoplasmic proteins might influence the properties of the glass [31]. In parallel, the desiccation metabolome contains the full set of building blocks known to assemble into sucrose-centered NaDES [103,104,105,106], which we hypothesize could form a localized liquid phase that sustains residual metabolism during late drying and early rehydration before the matrix finally vitrifies. Both extend the four-sugar picture rather than replace it.

The glassy state is moreover only the physical endpoint of a much wider tolerance program. The accumulation of the sugars that build the glass is itself controlled by the drought-responsive regulatory network [7,12]. Abscisic acid is a key regulator of desiccation tolerance, and in seeds it acts through the transcription factors abscisic acid-insensitive 3 and 5, which control the maturation program that includes the expression of LEA genes and the acquisition of desiccation tolerance [13]. Transcription factors also act directly on the glass-forming pathways: in the resurrection plant *Boea hygrometrica*, a WRKY transcription factor binds a W-box element in the promoter of the *GALACTINOL SYNTHASE* gene and induces the synthesis of raffinose and other RFOs [12], and in apple fruit, MdWRKY126 promotes sucrose accumulation by increasing the activity of sucrose-phosphate synthase and the expression of its gene and of a sucrose transporter gene [9]. The transcriptional changes in the sucrose and RFO pathways described here for *Haberlea* are therefore likely to be the downstream output of such regulators, whose identity in this species remains to be established. Alongside the glass, resurrection plants deploy protective mechanisms that are partly constitutive (high activities of antioxidant enzymes, high levels of anthocyanins and other polyphenols, and LEA transcripts already present under well-watered conditions) and partly induced on drying [12], while their membranes are protected through changes in composition and efficient antioxidant systems [13]. Vitrification complements rather than replaces these mechanisms: antioxidant systems limit oxidative damage while the cytoplasm is still fluid, whereas the glass, once formed, arrests the diffusion on which further damage depends. Because cytoplasmic glasses are complex mixtures of non-reducing sugars, proteins, organic acids, amino acids and salts whose composition likely varies from species to species [13], a natural next step is to understand how this regulatory network sets the composition of the glass in each species.

These principles also have a practical dimension. Genes from resurrection plants can improve stress tolerance in other species: the *GALACTINOL SYNTHASE* gene of *B. hygrometrica*, which encodes the first enzyme of RFO synthesis, made transgenic tobacco more tolerant of drought, and a MYB transcription factor from *C. plantagineum* increased the drought and salt tolerance of *Arabidopsis* [12]. A single gene, however, is unlikely to confer desiccation tolerance, and a more integrated approach is probably needed [12]. Vitrification itself is not a goal for crops, whose value depends on remaining hydrated and metabolically active. Yet the glass-forming sugars and their precursor galactinol also protect hydrated cells: both galactinol and raffinose protect against oxidative damage and sucrose can stabilize membranes [12], which may explain why *GALACTINOL SYNTHASE* improved the drought tolerance of tobacco without the need for vitrification. Other candidates are the molecules that protect cellular structures as water is lost, such as small heat shock proteins, which prevent protein aggregation and denaturation [13], and LEA proteins. LEA proteins are functionally diverse. Some act as molecular shields that prevent the aggregation of other proteins during drying and freezing [98]; others act on membranes, such as an α-helical LEA protein of the bdelloid rotifer *Adineta ricciae* that does not protect against protein aggregation, but interacts with dry phospholipid membranes [99]; and others stabilize sugar glasses [100] or the glassy state of seeds [102]. Some combine these roles: COR15A of *Arabidopsis*, a protein of the LEA_4 family, interacts with chloroplast membrane lipids in the dry state and also raises the T_g_ of dry sucrose glasses [101]. In transgenic tobacco, LEA proteins from *B. hygrometrica* helped the plants retain more water, maintain photosystem II activity and limit membrane damage under drought [12]. By distinguishing the LEA proteins and other molecules that build or reinforce the glass from those that shield enzymes and membranes during drying, a mechanistic understanding of desiccation tolerance can help to identify the right candidates for improving the drought tolerance of crops [13].

Set against orthodox seeds, the comparison that motivated this work can now be extended to the property that vitrification was always meant to explain. Like the seed cytoplasm [19], the *Haberlea* matrix can be described as a sugar glass with a definable composition, a modeled glass-transition surface and a maximally freeze-concentrated endpoint. It differs mainly in emphasis: the seed’s protective program is deployed once, at maturation, whereas the resurrection plant redeploys it repeatedly in vegetative tissue and tunes the sugar “recipe” to a habitat where drying may arrive as heat or as frost.

Vitrification is also exploited deliberately for preservation. The conditions under which cryoprotectant solutions vitrify can be represented on a phase diagram of the cryoprotectant–water system, while cryopreservation research takes inspiration from nature, exploring approaches based on extremophiles [108]. Sugars that vitrify on drying, such as sucrose and trehalose, also stabilize dried biomolecules and liposomes and inhibit protein denaturation during freeze-drying [49,57], and the stability of amorphous pharmaceutical solids is governed by their T_g_, which can be predicted from composition and water content with the same Gordon–Taylor relationship used here [109]. A state diagram built on the composition of a natural glass that, once dry, withstands even liquid-nitrogen temperatures could therefore help to choose the sugars, and their proportions, for formulations intended for the dry and cryogenic preservation of plant germplasm and other biological material.

Several limitations bound these conclusions. The sugar composition on which the model rests is drawn from chromatography, and no single platform—including the LC–MS adopted here—returns exact absolute concentrations: oligosaccharides can be partly degraded during sample preparation [110], and LC–MS quantification further depends on calibration, on analyte recovery and on matrix effects such as ion suppression; the aim, however, was not perfect quantification, but a physically coherent account of vitrification and of the building blocks that form the glass, for which the relative composition and its direction of change suffice. The state diagram rests on parameters derived from calorimetric data for sucrose and raffinose, but on modeled values for the minor oligosaccharides, and the supersaturated zone is inferred rather than directly observed. The cross-study transcriptomic concordance is an agreement among independent datasets, not a replicated meta-analysis, because the sources report no replicate-level variance; and both the near-infrared reinterpretation and the NaDES-assembly proposal rest on compositional and spectral correspondence rather than direct measurement. The heat-of-wetting assay was a simple, non-adiabatic setup that supports the qualitative result, but does not yield quantitative thermodynamic values. Similarly, although the convergence of our transcriptomic, metabolomic and biophysical evidence strongly suggests that the exotherm measured here reflects the amorphous glass of *Haberlea*, an exothermic response on rewetting a dried material should not in itself be taken as conclusive proof of a glassy state: biological tissues are chemically diverse, and in nature such a heat release cannot in every case be attributed with full certainty to an amorphous glass. These amount to a set of testable predictions. Calorimetry, vibrational and NMR spectroscopy of the dried matrix, and imaging mass spectrometry of sucrose-centered adducts would establish the glass transition and any eutectic phase directly. Near-infrared and FTIR spectra of anhydrous amorphous glasses of the same sugar composition and of dried tissue equilibrated at a series of low water content would show whether the S1 “water-dimer” band arises from non-bonded sugar hydroxyls, from singly bound surface water or from genuine water dimers. In situ enzyme and transcription assays across the drying trajectory would show whether activity is genuinely retained at low water content; and applying the same approach to further resurrection species would test how general the four-sugar, sucrose-dominated design proves to be.

## 4. Materials and Methods

### 4.1. Construction of the Supplemented State (Vitrification) Diagram of the Haberlea rhodopensis Sugar System

The supplemented state diagram summarizes the physical states of the *Haberlea rhodopensis* soluble-sugar fraction as a function of sugar concentration (% *w*/*w*, *x*-axis) and temperature (°C, *y*-axis). The soluble-sugar fraction was modeled as a four-component blend reflecting the measured composition of desiccated tissue: sucrose 91.9%, raffinose 7.3%, stachyose 0.38% and verbascose 0.45% (*w*/*w* of total soluble solids; calculated from the calibrated LC–MS data of Gechev et al. [34]). Each boundary of the diagram was constructed as described below. Where calorimetric data were unavailable (stachyose, verbascose), parameters were estimated from established polymer/oligomer models and are labeled “calc.” in the figure. Unless stated otherwise, temperatures were handled in kelvin for all calculations. The “strong/fragile” glass-former classification underlying the steep T_g_/viscosity dependence depicted in the diagram follows the general framework of Angell [14].

#### 4.1.1. Sugar Composition and Component Mass Fractions

The mass fractions of the glass-forming sugars were derived from the calibrated LC–MS sugar quantification of desiccated *Haberlea* tissue in the 2013 metabolome dataset [34], in which each sugar was quantified against authentic standards; in the supplementary data, the values are given as *w*/*w* % of the frozen sample (three biological replicates per condition, each analyzed in three injections), without the sample dry weights. Because all sugars were quantified in the same samples, their mass fractions do not depend on the weight basis. The four vitrification sugars together accounted for 99.4% (*w*/*w*) of the total quantified sugar pool; the residual fructose, glucose and galactinol (<1% combined) were minor mono- and precursor sugars and were excluded. Expressed as fractions of this four-sugar glass-former set, the mass fractions were sucrose 91.9%, raffinose 7.3%, stachyose 0.38% and verbascose 0.45% (Table S2), and these set the blend composition used throughout the state-diagram construction. A sensitivity analysis showed that the model outputs are robust to the exact sucrose:raffinose ratio: shifting it from 92:7 to 85:15 raises the dry-blend T_g_ by ~3 °C (65.6 → 68.7 °C) and changes T_g_′ by less than 1 °C (−42.0 → −41.1 °C), while C_g_′ (~80% solids), the vitrification point at 22 °C (~94% solids) and the freeze-drying endpoint (~13.5% water) remain essentially unchanged (Table S2).

Fold changes between the hydrated and desiccated states require a common weight basis, because the two tissues differ several-fold in water content. As the GC–MS data of the same samples are normalized to dry weight [34], the LC–MS log_2_ fold changes were rescaled to them by a single additive constant c (log_2_FC(scaled) = log_2_FC(LC–MS) + c), on the premise that a change of weight basis shifts the log_2_ fold changes of all compounds of a sample equally. c was estimated as the median of the differences log_2_FC(GC–MS) − log_2_FC(LC–MS) for the five sugars measured by both methods (sucrose, raffinose, galactinol, glucose and fructose; individual differences −0.95 to −2.35), giving c = −1.087 (a factor of 0.47). This median-based scaling was developed on the principle of probabilistic quotient normalization, which corrects dilution differences between samples by the median of the quotients of their signals [111]. Where a GC–MS dataset lists two peaks for the same sugar (derivatization isomers of fructose [34,36] and glucose [36]), their values were summed. The rescaled values therefore inherit the normalization of the GC–MS data; the calculation and the sensitivity of the results to c are given in Table S2. For the cross-study comparison (Figure 3), each fold change was calculated between the desiccated endpoint and the control of the respective study: Gechev et al. [34], desiccation (T3) versus the unstressed control (T1); Moyankova et al. [36], fully desiccated leaves (D3, RWC about 6%) versus the hydrated control (C); and Djilianov et al. [37], 168 h of drying (RWC 12%) versus the 0 h control (RWC 92%).

#### 4.1.2. Component Parameters

The T_g_ and Gordon–Taylor constant (k) used for each component are listed in Table 1. Sucrose and raffinose values are derived from calorimetric measurements; stachyose and verbascose values are modeled (Section 4.1.5 and Section 4.1.6).

#### 4.1.3. T_g_ Curves—Couchman–Karasz Equation

The concentration dependence of the T_g_ of each sugar–water system (the dashed pure-sugar reference curves and, via Section 4.1.4, the solid blend curve) was described by the Couchman–Karasz equation [24,66,112,113], which predicts the glass transition of a mixture from a thermodynamic (entropic) argument:

(1) ln T_g_ = (w_1_·ΔC_p,1_·ln T_g,1_ + w_2_·ΔC_p,2_·ln T_g,2_)/(w_1_·ΔC_p,1_ + w_2_·ΔC_p,2_)

where w_1_ and w_2_ are the mass fractions of sugar (solids) and water, T_g,1_ and T_g,2_ their T_g_ values, and ΔC_p,1_ and ΔC_p,2_ the C_p_ increments at the glass transition. In the linearized form used here it reduces to the compact working expression:

(2) T_g_ = (w_1_·T_g,1_ + k·w_2_·T_g,2_)/(w_1_ + k·w_2_), k = ΔC_p,2_/ΔC_p,1_

This expression is identical in form to the Gordon–Taylor expression, with the constant k identified as the ratio of C_p_ increments. The glass transition of pure water was taken as T_g,2_ = −135 °C [114]. For sucrose, T_g_ = 62 °C was measured and k = 4.70 was obtained by fitting Equation (2) to measured T_g_ values of sucrose solutions [114]; for raffinose, T_g_ = 103.25 °C and k = 5.386 were both obtained by fitting the same equation to measured T_g_ values of raffinose–water mixtures (with T_g_ of water taken as 135 K) [24].

#### 4.1.4. Glass Transition of the Four-Sugar Blend—Multicomponent Couchman–Karasz

For the four-sugar solid the blend glass transition was obtained from the multicomponent form of the same Couchman–Karasz treatment [113], each component contributing in proportion to its mass fraction and C_p_ increment:

(3) T_g,blend_ = Σ(w_i_·ΔC_p,i_·T_g,i_)/Σ(w_i_·ΔC_p,i_)

Because per-component heat-capacity increments were not separately available, the mixed solid was in practice parameterized as a single pseudo-component whose dry T_g_ and k were the mass-fraction-weighted means of the component values (T_g,blend_ = Σ w_i_ T_g,i_/Σ w_i_ ≈ 66 °C; k,blend = Σ w_i_ k_i_/Σ w_i_ ≈ 4.76); the blend T_g_-versus-water curve (solid blue line) was then computed from the working expression in Section 4.1.3 together with water (T_g_ = −135 °C). This parameterization reproduces the full multicomponent Couchman–Karasz result to within ~1 °C, under the assumption that the heat-capacity increments scale inversely with the Gordon–Taylor constants (ΔC_p,i_ = ΔC_p,w_/k_i_, from Equation (2)), a modeling choice rather than a measured property.

#### 4.1.5. Modeled Dry T_g_ of Stachyose and Verbascose—Fox–Flory Equation

Calorimetric T_g_ values are available for sucrose and raffinose, but not for the higher RFOs. Their dry glass transitions were therefore estimated from the Fox–Flory relationship [115] between T_g_ and chain length, expressed against the degree of polymerization (DP, the number of monosaccharide units: sucrose 2, raffinose 3, stachyose 4, verbascose 5):

(4) T_g_ = T_g,∞_ − K/DP

The two constants (T_g,∞_ and K) were calibrated on the experimentally derived sucrose (DP 2, 62 °C) and raffinose (DP 3, 103.25 °C) values, then used to predict stachyose (DP 4) and verbascose (DP 5), yielding T_g_ ≈ 124 °C and ≈ 136 °C respectively.

#### 4.1.6. Modeled Gordon–Taylor k of Stachyose and Verbascose—Simha–Boyer/Couchman–Karasz

The Gordon–Taylor constants of stachyose and verbascose (k = 5.69 and 5.87) were estimated by extrapolating along the homologous RFO series from the experimentally fitted raffinose value (k = 5.386), guided by the Simha–Boyer rule in the form applied to the Gordon–Taylor equation by Hancock and Zografi [109], in which k scales directly with the product of density (ρ) and T_g_ of the components, with T_g_ in kelvin:

(5) k ≈ (ρ_1_·T_g,1_)/(ρ_2_·T_g,2_)

The trend was cross-checked with the Couchman–Karasz relation [113], in which k equals the ratio of the C_p_ increments at T_g_:

(6) k = ΔC_p,2_/ΔC_p,1_

Extrapolating these parameters along a homologous series carries an inherent margin of error. Because stachyose and verbascose together make up less than 1% of the blend, however, this uncertainty has a negligible effect on the model: varying their dry T_g_ by ±30 °C and their k by ±1 changes the dry-blend T_g_ by at most ±0.25 °C and T_g_′ by less than 0.1 °C.

#### 4.1.7. Solubility (Saturation) Curves—Sucrose and Raffinose

For each sugar able to crystallize, the saturation boundary was expressed on the total-solids axis. Because the *x*-axis is the concentration of all sugars combined, the total-solids concentration C (%, *w*/*w*) at which a given sugar i (present at mass fraction w_i of the solids) reaches saturation at temperature T is:

(7) C_i,sat_(T) = 100 · r_i_(T)/(w_i_ + r_i_(T)), r_i_(T) = S_i_(T)/(1 − S_i_(T))

where S_i(T) is the pure-component solubility expressed as a mass fraction and r_i the corresponding saturated solute-to-water mass ratio. This construction correctly places a minor component (e.g., raffinose, only 7.3% of the solids) at the total-solids concentration at which its own concentration in the remaining water reaches saturation—which is why, at 22 °C, raffinose saturates near 74% total solids and sucrose near 69%. Sucrose solubility S_i(T) was taken from standard tables [116]; raffinose solubility from Zhang et al. [117] (values reported for raffinose pentahydrate were converted to anhydrous raffinose by the factor 0.8484 = M(anhydrous)/M(pentahydrate)). The raffinose data span 0–30 °C (solid curve); the segment above 30 °C is an extrapolation (dashed).

#### 4.1.8. Ice-Melting (Liquidus) Curve—Freezing-Point Depression (Route 3)

The ice-melting (liquidus) curve (cyan) is the freezing-point-depression curve of the solution: as ice forms it excludes solute, so the unfrozen phase concentrates and its freezing point falls. Freezing-point depression is a colligative property (set by the total moles of solute per kg water), so the curve was derived from the well-characterized sucrose–water liquidus [118,119,120] and mapped onto the four-sugar blend on an equal-molality basis. The blend molality at total-sugar mass fraction w is:

(8) m = 1000·w/(M_eff_·(1 − w))

with the mass-weighted effective molar mass:

(9) M_eff_ = 1/Σ(x_i_/M_i_) ≈ 352 g/mol

where x_i is the mass fraction of sugar i within the solids and M_i its molar mass (sucrose 342.3, raffinose 504.4, stachyose 666.6, verbascose 828.7 g/mol). Each blend concentration was converted to the sucrose concentration of the same molality and the measured sucrose freezing point read there. This accounts for the mixture’s colligative particle count—the heavier RFOs contribute fewer particles per gram—while retaining the experimentally-observed non-ideality of the concentrated liquidus. The upper branch is the equilibrium ice liquidus; below the ice–sucrose eutectic (~−14 °C), it is the metastable freeze-concentration extension. Because the blend is 92% sucrose, the resulting curve lies at most about 1 °C above the pure-sucrose liquidus.

#### 4.1.9. Maximal Freeze-Concentration (T_g_′/C_g_′) and Unfreezable Water

The maximally freeze-concentrated glass point (T_g′_, C_g_′) was located as the intersection of the ice-melting (liquidus) curve (Section 4.1.8) and the blend T_g_ curve (Section 4.1.4), determined numerically [66]. This gave C_g_′ ≈ 80% solids and T_g_′ ≈ −42 °C. At this point, the remaining ~20% water is kinetically trapped and cannot crystallize (the “unfreezable” water fraction).

#### 4.1.10. Vitrification Endpoints (Processing Routes)

The composition at which the system vitrifies during a given process was read from the blend T_g_ curve as the concentration at which T_g_ equals the process temperature [66,112]. For isothermal drying at ambient (22 °C, Route 2) this gives vitrification at ≈ 94% solids (~6% residual water); for freeze-drying at −20 °C (Route 3b), at ≈ 86.5% solids (~13.5% residual water). Melt–quench (Route 1) follows the anhydrous composition cooled through the dry-blend T_g_ (≈ 66 °C).

#### 4.1.11. Supersaturated Zone

The shaded region between the saturation curves (Section 4.1.7) and the blend T_g_ curve (Section 4.1.4) marks the states in which the syrup is simultaneously supersaturated with respect to sugar and above its glass transition, and therefore able to crystallize. This zone is a theoretical construct: its boundaries are the calculated phase curves described above—themselves derived from measured single-component data and established mixing models rather than measured on the four-sugar mixture—and no crystallization kinetics were determined for the blend. It therefore indicates where crystallization is physically permitted, not where it was observed to occur. Above 0 °C it corresponds to the classical devitrification region of amorphous sugars; below 0 °C it is shown only qualitatively (left-bounded by the ice-melting curve, Section 4.1.8, right-bounded by the T_g_ curve), a region additionally beyond the range of the underlying solubility and T_g_ data, in which sugar crystallization is increasingly suppressed kinetically as viscosity rises toward T_g_′ (Section 4.1.9).

### 4.2. Transcriptome Datasets, Sugar-Metabolism Gene Set and Merging of Haplotig Gene Models

Sugar-metabolism genes were identified in the annotated *Haberlea rhodopensis* genome [35] by searching the gene descriptions (the names of the closest Swiss-Prot homologues) for twelve enzyme classes of the RFO, sucrose and trehalose pathways: *GALACTINOL SYNTHASE, RAFFINOSE SYNTHASE, STACHYOSE SYNTHASE, SUCROSE-PHOSPHATE SYNTHASE, SUCROSE-PHOSPHATASE, SUCROSE SYNTHASE, NEUTRAL/ALKALINE* and *ACID INVERTASES, TREHALOSE-6-PHOSPHATE SYNTHASE, TREHALOSE-6-PHOSPHATE PHOSPHATASE*, and *TREHALASE*. The matches were curated manually (one invertase inhibitor was excluded), giving 123 gene models. *NEUTRAL/ALKALINE INVERTASES* were divided into cytosolic and organellar (chloroplastic or mitochondrial) groups, and *ACID INVERTASES* were grouped as vacuolar/cell wall, according to the localization given in the name of the closest Swiss-Prot homologue; this is an annotation-based assignment, not a targeting prediction.

The genome assembly contains redundant haplotig gene models, in which the two alleles of one gene were assembled as separate copies on different scaffolds. So that each gene was counted once and handled identically in all three datasets, gene models were merged and counted as one gene when their full-length proteins were ≥98% identical (global alignment, identity relative to the longer protein), differed in length by no more than 5%, and lay on different scaffolds. Merged groups were formed as connected components and named after the lowest-numbered gene model. The 123 gene models represent 108 genes, 13 of which are represented by two or three gene models (Table S1). In every dataset, the expression of a gene was obtained by summing its copies separately for each condition before the fold change was calculated.

The two 2024 experiments [35] were the cold experiment (desiccation versus the normal-condition control) and the dark experiment (the photoperiod experiment of Gupta et al. [35]; desiccation under the light regime versus its light control; samples 30D_L_7D_Dr and 30D_L_7D_L). For each gene, the TMM-normalized mean expression values [121] published by the authors were summed over copies, and the desiccation response was calculated as log_2_[(D + 0.5)/(C + 0.5)], where D and C are desiccation and control expression and 0.5 is a pseudocount in TMM units. Differential expression was taken from the authors’ lists of desiccation DEGs for the same contrasts. Because significance cannot be recalculated for summed copies (replicate-level counts are not published), a gene was counted as differentially expressed if any of its copies was on the list.

The Gechev et al. (2013) desiccation transcriptome [34] was produced by Illumina sequencing (Genome Analyzer IIx, 2 × 76 bp reads) with de novo assembly, and is available as assembled contigs, each carrying read counts in four hydration states (unstressed, drought, desiccation, rehydration), without biological replicates. To place these contigs in the genome gene space, the 96,353 contigs were aligned with blastn (megablast; NCBI BLAST+ 2.17.0 [122]) against the *Haberlea rhodopensis* reference coding transcriptome (93,489 coding sequences, CDS) of the published genome [35], retaining hits at ≥85% identity—a deliberately sub-100% threshold that allows for sequencing and short-read assembly errors and for allelic differences between the sequenced plants and the reference genome. Hits were graded by identity and alignment coverage (high: ≥98% identity and ≥60% coverage; good: ≥95% and ≥40%; moderate: ≥85% and ≥30%; coverage = aligned length relative to the shorter of the two sequences, contig or CDS), and alignments covering less than 30% were excluded; the remaining 81,546 contigs (~85%) were assigned to the gene model of their best hit, 27,166 gene models in total (roughly three contigs per gene model, reflecting the fragmentation of the short-read assembly (average contig length 421 bp)). For each assignment, whether it was a reciprocal best hit (contig ↔ gene) was also recorded; this was used in a sensitivity analysis (Section 4.3). For each gene, the raw reads of all contigs assigned to any of its copies were summed per condition, and the desiccation response was calculated as log_2_[(d·Nu/Nd + 1)/(u + 1)], where u and d are the summed unstressed and desiccation reads and Nu and Nd are the total reads of all 96,353 contigs in each library; that is, desiccation counts were scaled to the unstressed library size and a pseudocount of one read was added. Because the dataset has no biological replicates (one sample per condition), no replicate-based significance test is possible, and genes with |log_2_FC| ≥ 1 are described as strongly changed. Of the 108 genes, 81 were recovered in the 2013 data. Gene annotations were those of the published genome [35].

### 4.3. Cross-Study Concordance Analysis

Concordance of the desiccation log_2_ fold changes of the sugar-metabolism genes across the three studies (2024 cold, 2024 dark, Gechev 2013; the 81 genes with a computable fold change in all three) was assessed with rank-based statistics, because none of the source studies report replicate-level *p*-values for a formal per-gene meta-analysis. The per-gene log_2_ fold changes were calculated as described in Section 4.2. Overall agreement was quantified by Kendall’s coefficient of concordance (W) [123], treating the three studies as raters and the 81 genes as items, with significance from the associated χ^2^ statistic; the Friedman test and pairwise Spearman rank correlations (ρ) served as complementary measures. Directional consistency—genes changing in the same direction in all three studies—was compared, using a one-sided exact binomial test, with the number expected if the three studies were independent, calculated from the observed proportion of up-regulated genes in each study, and genes with |log_2_FC| ≥ 1 and the same sign in all three studies were additionally classed as strongly consistent. Because co-regulated genes are not statistically independent, a sensitivity analysis treating each enzyme class as a single independent unit (each enzyme class represented by the mean log_2_FC of its genes in each study, with W calculated on the 12 class means and its significance assessed by permutation, the class means being permuted independently within each study; 200,000 permutations) and a cluster bootstrap resampling whole enzyme classes (5000 resamples; 95% percentile interval) were additionally performed; the concordance remained significant throughout (Table S1). In addition, the 2013 log_2_FC values were recomputed using only contigs mapped at ≥95%, ≥98% or 100% identity, and the analysis was repeated with these values and for the genes all of whose contig assignments are reciprocal best hits, to test whether the concordance depends on the permissive 85% mapping threshold. For comparison, the analysis was also performed on the 91 individual gene models with haplotig copies counted separately (Table S1). Analyses were carried out in Python 3.12 (SciPy 1.18, pandas 3.0; Biopython 1.88 for the protein alignments).

### 4.4. Software and Figure Preparation

All numerical calculations, statistical analyses and figures were produced in Python 3.12 (Python Software Foundation, Wilmington, DE, USA). Statistical methods are described in Section 4.9. Most figures—the cross-study concordance summary, the gene- and metabolite-response plots and the composition figures were drawn with Matplotlib 3.11 (NumPy 2.5); the state (vitrification and temperature) diagrams were composed as scalable-vector graphics and rasterized at 300 dpi for publication.

### 4.5. Cryotolerance of Desiccated Haberlea rhodopensis

*Haberlea rhodopensis* plants were grown as described for the cold experiment in the reference genome study [35]. Desiccated plants (~10% RWC) were exposed to −80 °C for three days (*n* = 6) or immersed in liquid nitrogen (−196 °C) for 8 min (*n* = 4), alongside a desiccated control held at room temperature (*n* = 4). As hydrated controls, fully turgid plants (*n* = 4 per freezing treatment) were frozen identically. All plants were rehydrated the following day and scored for survival approximately 24 h later. The desiccated-frozen and hydrated-frozen groups were compared with the two-tailed Fisher’s exact test, and survival proportions are reported with Wilson 95% confidence intervals. Freezing treatments of desiccated *H. rhodopensis* at −20, −80 and −196 °C, combined with electrolyte leakage and chlorophyll fluorescence measurements, are also used, with the same protocol, in an independent study addressing a different question.

### 4.6. Cryotolerance of Desiccated Xerophyta elegans Seedlings

*Xerophyta elegans* seeds were provided by Prof. Nicola Illing (University of Cape Town, South Africa). Seeds were visually inspected and sorted into batches of 100. Each batch was heat-treated at 65 °C for 1 h and then treated sequentially, with vortexing, in 100% ethanol (10 s), 70% ethanol containing 1 µL mL^−1^ Triton X-100 (20 s) and 15% H_2_O_2_ containing 1 µL mL^−1^ Triton X-100 (1 min). Seeds were then washed three times by inversion in distilled water (dH_2_O) and transferred onto sterile filter paper to dry. Each batch was plated on half-strength Murashige and Skoog (MS) agar medium, sealed with Parafilm and transferred to a plant growth chamber at 23–24 °C under a 16/8 h light/dark cycle to germinate.

For the freezing-tolerance assay, seedlings aged 30–37 days were arranged in petri dishes (20 seedlings per dish) on Whatman filter paper (Cytiva, Maidstone, UK) moistened with 2 mL dH_2_O. The dishes were closed, but not sealed with Parafilm to permit slow drying, and the seedlings were left to desiccate for seven days. Desiccated seedlings were then assigned to one of four treatments (three dishes, 60 seedlings, per condition): an unfrozen control held at 23–24 °C in the growth chamber; −20 °C and −80 °C for 72 h; and −196 °C by immersion in liquid nitrogen for 1 h. After treatment, all dishes were returned to the growth chamber at 23 °C for 24 h and then rehydrated. Survival was scored seven days after rehydration as the percentage of seedlings that resumed growth (*n* = 60 per condition). Differences in survival among conditions were tested with the Fisher–Freeman–Halton exact test, and proportions are reported with Wilson 95% confidence intervals.

### 4.7. Cryotolerance of Desiccated Selaginella lepidophylla

*Selaginella lepidophylla* plants were obtained from a commercial online supplier (bilki.bg, Bulgaria; lot L25); the plants were imported and originate from Mexico. Before treatment, the RWC of the air-dry plants was determined on 40 samples (S1–S40), each consisting of three frond tips of about 2.5–4 cm taken from an air-dry plant; these sample numbers are not linked to the plant numbers (P1–P40) assigned when the plants were allocated to treatments and used for all later measurements (Table S4). Air-dry plants (*n* = 10 per condition) were assigned to four treatments: an unfrozen control kept at room temperature (22–23 °C); −20 °C or −80 °C for 48 h; or immersion in liquid nitrogen (−196 °C) in an open container, in which the plants were fully submerged for ~1.5 h and left until the nitrogen had evaporated (~3.5 h). The control and liquid-nitrogen groups were rehydrated 24 h after treatment, and the −20 °C and −80 °C groups 24 h after removal from the freezers. Survival was scored as unrolling and greening of the plant after rehydration and documented photographically (Figure S2a). RWC and electrolyte leakage were determined 72 h, and Fv/Fm 216 h, after rehydration in each group (treatment calendar in Table S4). Two plants of the liquid-nitrogen group (P1, P4) had been used in a preliminary test in which they were additionally immersed in liquid nitrogen while hydrated; they were excluded from the Fv/Fm analysis, but retained for RWC and electrolyte leakage, which had been measured before that test.

For the partially rehydrated reference, the ten control plants were used six days after rehydration. They were matched in pairs by their electrolyte leakage 72 h after rehydration and by visual comparison (P11–P13, P12–P18, P14–P19, P15–P17, P16–P20). Two plants (P12 and P13) were then excluded because a severe fungal infection had damaged their tissue, and their partners were paired with each other (P11–P18). In each of the four pairs, one plant, chosen by coin flip (P17–P20), was immersed in liquid nitrogen as above, while the other remained unfrozen. RWC and Fv/Fm were measured immediately before freezing, and Fv/Fm 16 h and 72 h afterwards; for this RWC measurement, two fronds per plant were taken instead of three, to preserve tissue for the subsequent measurements.

All other RWC measurements and all electrolyte leakage measurements were made on three fronds per plant, one taken at random from each of three regions of the rosette (center, middle ring and outer ring; the outermost, oldest fronds were not sampled; Figure S2b). RWC was calculated [124] as:

(10) RWC = (FW − DW)/(TW − DW) × 100

with fresh weight (FW) at sampling, turgid weight (TW) after 24 h in deionized water (tube filled to 40 mL) and dry weight (DW) after 48 h at 65 °C. For electrolyte leakage, the fronds were weighed and incubated for 24 h in deionized water in a tube filled to 30 mL; electrical conductivity (EC) was measured with a conductivity meter (HI8733N, Hanna Instruments, Woonsocket, RI, USA), the samples were boiled in a water bath for 45 min and measured again (EC_100_). Electrolyte leakage (EL, %) was calculated following the method described in [125], as:

(11) EL = (EC − EC_blank_)/(EC_100_ − EC_blank_) × 100

with EC_blank_ = 1.7 µS cm^−1^.

Chlorophyll fluorescence was imaged with a FluorCam 800MF controlled by FluorCam 7 software (version 1.0.15.0; Photon Systems Instruments, Drásov, Czech Republic) after 45 min of dark adaptation (actinic lights 1 and 2 at 50%, saturating pulse at 80%, detector sensitivity 20%). Fo, Fm and Fv/Fm [126] were obtained for each plant from circular regions drawn over the plant, counting only pixels above the fluorescence threshold. One measurement in which the geometry of the partly curled rosette did not allow the plant to be captured adequately (P15, 16 h after liquid nitrogen; Fv/Fm 0.10) was excluded from the analysis. As references, fronds were taken at random from different experimental plants after all measurements had been completed: healthy-looking green fronds, which were measured directly and after being killed by autoclaving (121 °C), and dead-looking brown fronds, which were dried at 105 °C overnight; all were measured in the same way, and without a sample the instrument recorded no signal.

### 4.8. Exothermic Hydration of the Desiccated Glassy Matrix

Rewetting of a desiccated material releases heat if it contains an amorphous glass, because hydration of an amorphous sugar matrix is exothermic whereas dissolution of the corresponding crystalline sugar is endothermic [74]; we used this contrast as a functional test for a glassy matrix. Tomato (*Solanum lycopersicum* “Moneymaker”; seeds from 123zaden.nl, The Netherlands) plants were grown for 20 days; one hydrated plant provided the hydrated-tissue samples and three plants were air-dried outdoors at 33–35 °C for the desiccated-tissue samples. *Haberlea rhodopensis* was grown in vitro as described previously [127]; two hydrated plants provided the hydrated samples and five plants were air-dried on the growth-chamber shelf at 22 °C for the desiccated samples. For the desiccated material the whole plant was used, including the roots; culture medium was rinsed from the *Haberlea* roots before drying and soil was mechanically removed from the tomato roots before grinding. Avocado (*Persea americana*) fruits, containing a recalcitrant (desiccation-sensitive) seed, were purchased from a local supermarket as a non-glass-forming control, and tomato seeds (“Moneymaker,” as above) served as an orthodox, desiccation-tolerant seed control.

Matched amorphous and crystalline sugar standards of identical composition (approximately 80% sucrose, 20% glucose, *w*/*w*) were prepared from D-glucose monohydrate (Duchefa Biochemie, Haarlem, The Netherlands; G0802) and sucrose (Duchefa S0809). The crystalline standard was a physical mixture of the dry sugars in a microcentrifuge tube (exact masses in Table S5). The amorphous glass was prepared by melt-quenching [54]: 5.6 g sucrose, 1.4 g glucose and 3 mL water were heated with slow stirring on a hotplate in a fume hood while the temperature was monitored continuously, with the glucose serving as a crystallization inhibitor. Stirring was stopped once no sucrose crystals remained, and at ~150 °C the melt was poured immediately as a thin layer onto aluminum foil lining a metal vessel that had been pre-chilled at −20 °C for 1 h and held on ice, quenching it to a transparent glass. Its amorphous state was not verified instrumentally (e.g., by DSC or polarized-light microscopy), and a small crystalline fraction may have remained. Because dissolution of crystalline sugar is endothermic, such a fraction would only partly offset the heat released by the amorphous portion; the clearly exothermic response of the glass therefore indicates that it was predominantly amorphous.

The differently dried materials, together with the sugar standards, were then equilibrated under identical conditions (35 °C, 48 h) before measurement; this common end-point served to standardize all desiccated samples to a reproducible, equilibrated low-moisture state. All materials except the avocado seeds were ground to a fine powder with a mortar and pestle at room temperature. The big avocado seeds were ground in a coffee/nut grinder. To compare hydrated and desiccated tissue on a similar dry-matter basis, the water content of both the fresh and the desiccated tomato and *Haberlea* material was determined gravimetrically (sample versus oven-dry mass; Table S6). Dry weights were obtained by drying overnight in an oven at 65 °C and re-weighing after a further hour, the value being taken as final once successive weighings agreed (constant weight). Fresh tissue had dry-matter fractions of ~15% and ~13% (tomato and *Haberlea*, respectively), whereas the desiccated material used in the assay retained only ~5% residual moisture (~95% dry matter; ~5.3% and ~4.6%, respectively), confirming a fully desiccated state. The mass of hydrated tissue assayed (~600–750 mg) was chosen so that its dry-matter content (~80–115 mg) approximated that of the desiccated samples (about 100 mg; dry matter ~52–109 mg).

Each weighed sample was placed in a 1.5 mL microcentrifuge tube and equilibrated for 5 min in a dry-block thermostat (HB150-S2, DLAB Scientific, Beijing, China) set to 27.5 °C (room temperature 23–26 °C). Temperatures were read with a portable digital thermometer (Isolab Laborgeräte, Eschau, Germany; cat. 060.01.001; resolution 0.1 °C). Deionized water (conductivity 2.1 µS cm^−1^) was equilibrated in the same block. For each measurement the water was briefly stirred and its temperature recorded once stable for ~15 s (*T*_w_). The sample followed the same protocol—after short stirring, the temperature was recorded until stable for ~15 s (*T*_m_). A measured volume of the equilibrated water (27–50 µL; Table S5) was added to the sample, briefly stirred, and the extremal temperature recorded once no further change occurred over 15 s (*T*_mix_). The extremal temperature was reached within one to two minutes of wetting. Mixing and reading were performed with the tube kept in the block to avoid warming of the small sample by handling. The actual block-reported temperature of each component was recorded before every mixing rather than assuming the set point.

Because the water and the material were not always at the same temperature, the temperature change was referenced to the expected no-reaction mixing temperature obtained from the method of mixtures:

(12) T_exp_ = (m_m_c_m_T_m_ + m_w_c_w_T_w_)/(m_m_c_m_ + m_w_c_w_)

where m, c and T denote mass, specific heat and initial temperature and the subscripts m and w the solid material and the added water; c_w_ = 4.18 J g^−1^ K^−1^, m_w_ was taken from the added volume (1 µL ≈ 1 mg), and c_m_ was assigned literature values (≈1.3–1.6 J g^−1^ K^−1^ for desiccated tissue [128] and amorphous sugars [74], ≈1.24 for crystalline sucrose [129] and ≈3.9 for hydrated tissue [128]). The exothermic response was expressed as ΔT = T_mix_ − T_exp_, with positive values indicating heat release. Because the two components were pre-equilibrated to within 0.5 °C (mean difference 0.17 °C), T_exp_—which is bounded between the two initial temperatures—depends only weakly on the assumed cm: varying c_m_ by ±15% changed ΔT by at most 0.02 °C (0.006 °C for hydrated tissue); even a ±50% change altered it by at most 0.033 °C, far below the differences between groups. Because the reaction proceeded inside the block heater, whose large thermal mass stayed near the 27.5 °C set point, heat would be exchanged with the sample whenever it departed from that temperature—from the sample into the block when an exothermic sample warmed above it, and from the block into the sample when an endothermic sample cooled below it. This should damp the excursion in either direction, so the recorded ΔT most likely underestimates the true (adiabatic) magnitude for both exothermic rises and endothermic dips rather than overstating it, though we did not quantify the effect. This setup was arranged as a simple proof-of-concept assay. The system was not adiabatic: the dry block acts as a large heat sink, and heat exchange with the tube, the thermometer probe and the surroundings (including evaporation) was neither controlled nor quantified. The absolute ΔT values are therefore not reliable measures of the heat of hydration, and no thermodynamic quantities (e.g., enthalpies) were derived from them. The assay is interpreted qualitatively through the sign of ΔT and its comparison between groups measured with the same apparatus and protocol; quantitative values would require isothermal calorimetry or DSC.

Group Δ*T* values were compared with the Kruskal–Wallis test, followed by planned pairwise comparisons (desiccated versus hydrated tissue; amorphous versus crystalline sugar; orthodox versus recalcitrant seed; orthodox seed versus dried vegetative tissue of the same species; and desiccation-tolerant versus desiccation-sensitive dried tissue) using the exact Mann–Whitney U test with Holm correction and Hedges’ g effect sizes (with small-sample correction); the association between physical state and exothermic response was assessed by point-biserial correlation between glass-forming materials and non-glass-forming biological materials (the crystalline sugar standard excluded) and by ordinal (rank) correlation across the three physical states (crystalline < non-glass < glass). As a sensitivity check, both correlations were also computed for the biological materials alone. Analyses were performed in Python with SciPy.

### 4.9. Statistical Analysis

All analyses were performed in Python 3.12 with SciPy 1.18 [130] and pandas 3.0. Unless stated otherwise, tests were two-sided and *p* < 0.05 was considered significant. Values are given as mean ± SD, the unit of analysis is the individual biological sample (plant, seedling or tissue sample), and *n* is stated for each experiment. The data needed to repeat the analyses are provided in Tables S1–S6.

#### 4.9.1. Cross-Study Transcriptome Concordance

Because the source studies report no replicate-level variance, per-gene tests of differential expression and false-discovery-rate control could not be applied; agreement among the three datasets was therefore assessed on the log_2_ fold changes of the 81 genes (Table S1) with rank-based statistics. Kendall’s coefficient of concordance W was calculated with the three studies as raters and the genes as items (with correction for ties), and its significance was obtained from χ^2^ = m(n − 1)W with n − 1 degrees of freedom (m = 3 studies, n = number of genes). The Friedman test (genes as blocks, studies as treatments) and pairwise Spearman correlations served as complementary measures. Directional agreement, the number of genes changing in the same direction in all three studies, was tested with a one-sided exact binomial test against the probability p_0_ = Πp_i_ + Π(1 − p_i_), where p_i_ is the proportion of up-regulated genes in study i, i.e., the agreement expected if the studies were independent. To allow for the non-independence of co-regulated genes, each enzyme class was represented by the mean log_2_FC of its genes in each study and W was calculated on the 12 class means; its significance was obtained by permuting the class means independently within each study (200,000 permutations; *p* = (k + 1)/(N + 1), where k is the number of permutations with W at least as large as observed and N the number of permutations). In a cluster bootstrap, the 12 classes were resampled with replacement 5000 times, W and the fraction of directionally concordant genes were recalculated on the pooled genes of each resample, and the 2.5th and 97.5th percentiles are reported.

#### 4.9.2. Cryotolerance

For *Haberlea*, survival of desiccated and hydrated plants was compared with the two-tailed Fisher’s exact test on the 2 × 2 table of surviving and dead plants, separately for each freezing treatment and for the counts summed over both treatments; survival proportions are reported with Wilson score 95% confidence intervals [131]. Survival of *Xerophyta* seedlings across the four temperature conditions was compared with the Fisher–Freeman–Halton exact test [132] on the 2 × 4 table, the *p*-value being obtained by complete enumeration of all tables with the observed margins. For *Selaginella*, survival across the four conditions was compared with the Fisher–Freeman–Halton exact test on the 2 × 4 table and is reported with Wilson 95% confidence intervals. RWC and electrolyte leakage (72 h after rehydration; *n* = 10 per group) and Fv/Fm (216 h after rehydration; *n* = 4 in the control, 10 at −20 °C and −80 °C and 8 after liquid nitrogen) were compared among the four groups with the Kruskal–Wallis H test (tie-corrected, χ^2^ approximation), and the association between RWC and electrolyte leakage across all 40 plants with Spearman’s rank correlation. In the partially rehydrated reference, Fv/Fm at 72 h and its change from before freezing to 72 h were compared between the four plants immersed in liquid nitrogen and their four unfrozen partners with the exact two-sided Mann–Whitney U test; the same test was used to compare Fv/Fm of these plants at 72 h with that of the desiccated liquid-nitrogen group (*n* = 8). The excluded measurements are described in Section 4.7, and all individual values are given in Table S4. Wilson 95% confidence intervals are shown for all groups in Figure 7.

#### 4.9.3. Heat of Wetting

For each sample, ΔT was calculated as the measured temperature of the mixture minus the expected no-reaction mixing temperature (ΔT = T_mix_ − T_exp_, with T_exp_ from Equation (12)). The eight groups were compared with the Kruskal–Wallis test, followed by the five planned pairwise comparisons listed in Section 4.8, using the exact Mann–Whitney U test with Holm correction across the five comparisons. Effect sizes are Hedges’ g = J(x̄_1_ − x̄_2_)/s_p_, where s_p_ is the pooled standard deviation and J = 1 − 3/(4(n_1_ + n_2_) − 9) the small-sample correction [133]. For the full matrix of 28 pairwise comparisons (Figure S3), exact Mann–Whitney *p*-values were corrected with the Benjamini–Hochberg procedure. The association between physical state and ΔT was quantified on individual samples by point-biserial correlation between glass-forming materials (desiccated *Haberlea*, the amorphous sugar glass and orthodox tomato seeds; coded 1) and non-glass-forming biological materials (hydrated *Haberlea* and tomato tissue, dried tomato vegetative tissue and the recalcitrant avocado seed; coded 0), the crystalline standard being excluded, and by Spearman rank correlation across three ordinal states assigned as above (crystalline = 0, expected non-glass = 1, expected glass = 2). The glass or non-glass status of each material was assigned a priori from its expected physical state, independently of the measured ΔT: orthodox seeds and desiccated tissues of desiccation-tolerant plants are expected to form intracellular glasses on drying, whereas hydrated tissues are not, and desiccation-sensitive tissues, such as the recalcitrant avocado seed and dried vegetative tissue of tomato, generally lose viability before a glassy state is reached [19,20,21], although they may still vitrify when air-dried after losing viability [32]; the classification therefore reflects whether a glass forms in living tissue rather than excluding glassy regions in the dried material; the amorphous and crystalline sugar standards were classified by their mode of preparation. Both correlations were also calculated for the biological materials alone, with the two sugar standards excluded.

#### 4.9.4. Sugar Pools

Means ± SD of the LC–MS sugar amounts (per mass of the frozen sample) are based on the three biological replicates per condition of the 2013 LC–MS dataset, each the mean of three injections. No statistical tests were applied to these data.

#### 4.9.5. State-Diagram Model

The model is deterministic. Its robustness was assessed by sensitivity analyses of the sugar composition and of the parameters of the minor sugars (Table S2) rather than by statistical tests.

## 5. Conclusions

Integrating the available molecular data with solid-state biophysics and targeted experiments provides a composition-resolved and biophysically grounded description of the glassy matrix of a resurrection plant. Our analysis indicates that in *Haberlea rhodopensis*, this matrix is a sucrose-dominated, four-sugar galactosyl-sucrose glass, most likely reinforced by additional biomolecules such as LEA proteins, and holding a small, functionally bound water fraction. Its formation is governed by the removal of water rather than by temperature, so tolerance of freezing and even of liquid-nitrogen temperatures in the dried state is one expression of the plant’s tolerance of drying. Rather than a mere remnant, the residual water appears to be an integral, protective component of the living glass.

Taken together, the model sheds light on the mechanism of desiccation tolerance in resurrection plants, connecting the accumulation of protective sugars with the formation of the glass during drying, its stability in the dry state and its passage back to the liquid state on rehydration. Beyond resurrection plants, this framework links the composition of a biological glass, inferred from molecular data, to its predicted physical behavior, and it can be applied to other desiccation-tolerant systems, including orthodox seeds and pollen. Some of its predictions are supported by the experiments presented here, namely the survival of dried tissue at liquid-nitrogen temperature, consistent with the absence of freezable water, and the heat released on rewetting the dried matrix, consistent with its amorphous state. Others, such as the glass-transition temperature of the dry matrix and its freeze-concentration point, remain to be tested calorimetrically. Placing the published molecular, metabolic and spectroscopic data within a single physical model also allows observations that are difficult to interpret on their own to be re-examined: considered together with the composition of the glass that forms at the same time, the near-infrared “water-dimer” signal of dried *Haberlea* led us to propose that part of it arises from the non-bonded hydroxyls of the vitrifying sugars and from water bound singly to biomolecular surfaces, rather than from free water dimers, a proposal that near-infrared and FTIR spectroscopy of matched sugar glasses and dried tissue could test directly. Viewing vitrification as part of the wider drought-response network helps to separate the components that build the glass from the protective proteins, such as shield-type LEA proteins, that stabilize other proteins and membranes during drying. These protective proteins, together with sugars acting in the hydrated cell, rather than vitrification itself, are the more promising candidates for improving the drought tolerance of crops. Finally, a state-diagram model built on the sugar composition of a natural, desiccation-tolerant glass could inform the design of sugar-based formulations for the dry and cryogenic preservation of plant germplasm and other biological material.

## Figures and Tables

**Table 1:** Component parameters used to construct the state diagrams. For each glass-forming sugar and for water, the mass fraction of total solids, the dry-state glass-transition temperature (T_g_), and the Gordon–Taylor constant (k) are given, together with the basis or source of each value. Stachyose and verbascose values were modeled (Fox–Flory/Simha–Boyer relations); the final row is the mass-weighted four-sugar blend used in the state diagrams.

Component	Mass % of Solids	Dry T_g_ (°C)	k	Basis/Source
Sucrose	91.9	62	4.70	T_g_ measured, k fitted (Section 4.1.3)
Raffinose	7.3	103.25	5.386	T_g_ and k fitted to measured data (Section 4.1.3)
Stachyose	0.38	124	5.69	modeled (Section 4.1.5 and Section 4.1.6)
Verbascose	0.45	136	5.87	modeled (Section 4.1.5 and Section 4.1.6)
Water	—	−135	—	reference value (Section 4.1.3)
Blend (all sugars)	100	≈66	≈4.76	mass-weighted (Section 4.1.4)

## Data Availability

The transcriptomic and metabolomic datasets analyzed in this study are available in the published studies cited herein. The original contributions presented in this study are included in the article and its Supplementary Material (Tables S3–S6). The analysis and modeling code that supports the findings of this study is available from the corresponding author upon reasonable request.

## Supplementary Materials

The following supporting information can be downloaded at https://www.mdpi.com/article/10.3390/ijms27219405/s1.

## Abbreviations

The following abbreviations are used in this manuscript.

BET	Brunauer–Emmett–Teller
CDS	coding sequence
C_g_′	solids concentration of the maximally freeze-concentrated glass
CI	confidence interval
C_p_	heat capacity
DEG	differentially expressed gene
DP	degree of polymerization
DSC	differential scanning calorimetry
DW	dry weight
EC	electrical conductivity
EL	electrolyte leakage
EPR	electron paramagnetic resonance
FDR	false-discovery rate
FTIR	Fourier-transform infrared spectroscopy
Fv/Fm	maximum quantum yield of photosystem II
FW	fresh weight
GABA	γ-aminobutyric acid
GC–MS	gas chromatography–mass spectrometry
GH36	glycoside hydrolase family 36
HPAEC–PAD	high-performance anion-exchange chromatography with pulsed amperometric detection
LC–MS	liquid chromatography–mass spectrometry
LEA	late embryogenesis abundant
NaDES	natural deep eutectic solvent
NMR	nuclear magnetic resonance
RFO	raffinose family oligosaccharide
RWC	relative water content
SD	standard deviation
SnRK1	sucrose non-fermenting-1-related protein kinase 1
T6P	trehalose-6-phosphate
Tc	critical temperature
T_g_	glass-transition temperature
T_g_′	glass-transition temperature of the maximally freeze-concentrated solution
TMM	trimmed mean of M-values
TPP	*TREHALOSE-6-PHOSPHATE PHOSPHATASE*
TPS	*TREHALOSE-6-PHOSPHATE SYNTHASE*
TW	turgid weight
